# Current status, hotspots, and trends in cancer prevention, screening, diagnosis, treatment, and rehabilitation: A bibliometric analysis

**DOI:** 10.32604/or.2025.059290

**Published:** 2025-05-29

**Authors:** CHUCHU ZHANG, YING LIU, ZEHUI CHEN, YI LIU, QIYUAN MAO, GE ZHANG, HONGSHENG LIN, JIABIN ZHENG, HAIYAN LI

**Affiliations:** 1 Institute of Information on Traditional Chinese Medicine, Chinese Academy of Chinese Medical Sciences, Beijing, 100700, China; 2 Institute of Chinese Materia Medica, China Academy of Chinese Medical Sciences, Beijing, 100700, China; 3 Department of Traditional Chinese and Western Medicine, Shandong Cancer Hospital and Institute, Shandong First Medical University and Shandong Academy of Medical Sciences, Jinan, 250117, China; 4 Guang’anmen Hospital, Chinese Academy of Chinese Medical Sciences, Beijing, 100053, China; 5 Department of Cardiology, The First Affiliated Hospital of Zhengzhou University, Zhengzhou, 450052, China; 6 Oncology Department of Integrated Traditional Chinese and Western Medicine, China-Japan Friendship Hospital, Beijing, 100029, China

**Keywords:** Cancer prevention, Cancer screening, Cancer diagnosis, Cancer treatment, Cancer rehabilitation, Bibliometrics

## Abstract

**Objectives:**

Decades of clinical and fundamental research advancements in oncology have led to significant breakthroughs such as early screening, targeted therapies, and immunotherapy, contributing to reduced mortality rates in cancer patients. Despite these achievements, cancer continues to be a major public health challenge. This study employs bibliometric techniques to visually analyze the English literature on cancer prevention, screening, diagnosis, treatment, and rehabilitation.

**Methods:**

We systematically reviewed publications from 01 March 2014, to 01 March 2024, indexed in the Web of Science core collection. Tools such as VOSviewer Version 1.6.20 is characterized by its core idea of co-occurrence clustering. CiteSpace 6.3.R3 is distinguished by its powerful capabilities in bibliometric analysis, including co-citation analysis, co-occurrence analysis of keywords, author collaboration network analysis, and journal co-citation analysis, providing effective insights into research hotspots and detecting emerging trends. Bibliometrix version 3.0.3 offers rich visualization features, including collaboration network diagrams, citation distribution graphs, and keyword clouds. facilitated the analysis of the literature, helping to map out the current research landscape, identify pressing issues, and discern emerging trends, thus offering insights for future research directions.

**Results:**

The analysis revealed that major research hotspots include lung and breast cancer. Attention is predominantly concentrated on cancer treatment, subdivided into targeted therapy, immunotherapy, traditional Chinese medicine, and the development of new anticancer drugs. Significant terms identified in the study include immune checkpoint inhibitors, tumor microenvironment, and cancer stem cells.

**Conclusion:**

This bibliometric analysis highlights the evolving directions in oncology research, pinpointing nanotherapy, resistance to targeted therapies, and the integration of artificial intelligence as pivotal future research avenues in the prevention, screening, diagnosis, treatment, and rehabilitation of cancer.

## Introduction

The National Cancer Institute (NCI) recently released the latest “Cancer Statistics 2024” [1] in the esteemed international journal CA. According to this report, despite substantial advancements in surgical techniques, radiotherapy, targeted therapies, and immunotherapy, along with ongoing research breakthroughs that have slightly improved treatment outcomes, significant challenges remain [2]. These include reducing cancer incidence, preventing postoperative recurrence and metastasis, and extending patient survival. Malignant tumors continue to pose a grave threat to public health [3]. This study utilizes bibliometric methods to comprehensively review global publications from the past decade on cancer prevention, screening, diagnosis, treatment, and rehabilitation. The aim is to analyze the current research status, hotspots, and trends in oncology, seeks to guide future research directions and strategies, providing valuable references for innovative approaches to cancer management.

## Materials and Methods

### Data sources

Our bibliometric analysis used the Web of Science database (https://www.webofscience.com/wos/) (accessed on 25 December 2024), covering publications from 01 March 2014, to 01 March 2024.

### Inclusion criteria for literature

We employed a search strategy in English, focusing on the keywords TI = ((“tumor” OR “neoplasm” OR “cancer”) AND (“prevention” OR “diagnosis” OR “screening” OR “rehabilitation” OR “treatment”)). “TI” means Title. This targeted oncology-related literature within the core collection of the Web of Science.

### Exclusion criteria for literature

Excluded from our analysis were documents with incomplete information, conference papers, and technological reports.

### Statistical methods

**Figure 1:**
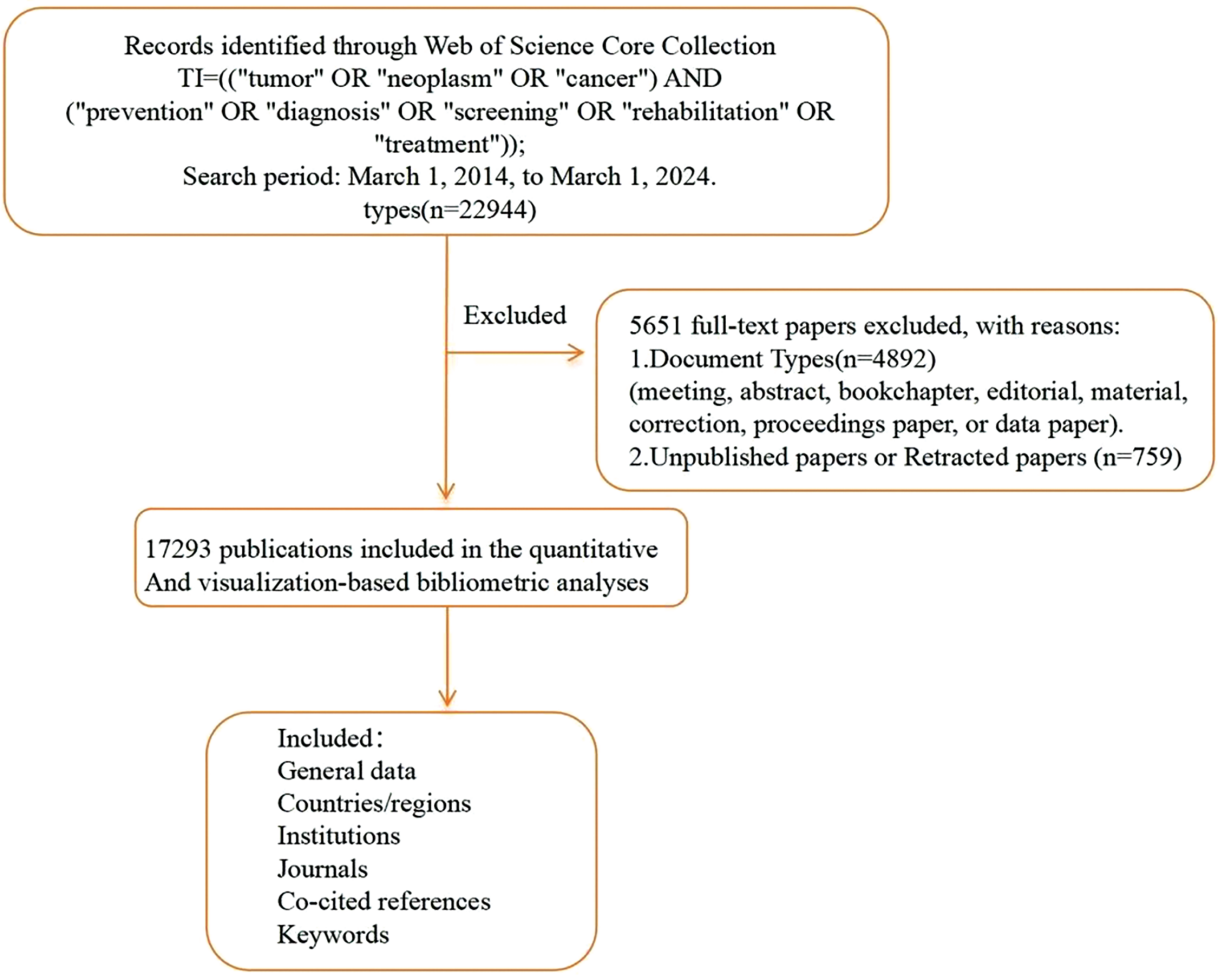
Literature screening.

The literature management software Endnote X9.1 v19.1.0.12691 was used to organize the collected data, which were then exported to Excel, including details such as titles, authors, affiliations, sources, keywords, abstracts, and publication dates. After deduplication, 17,293 English-language articles remained and were further analyzed. Fig. 1 extraction involved merging, comparing, and conducting an in-depth examination of these documents. Keyword decoding highlighted core research directions in global oncology. Co-linearity clustering of keywords identified key research hotspots, while a timeline analysis of keywords revealed evolving trends, helping to comprehensively delineate the main themes and development trajectories of cancer research.

## Results

### National annual publication trends

**Figure 2:**
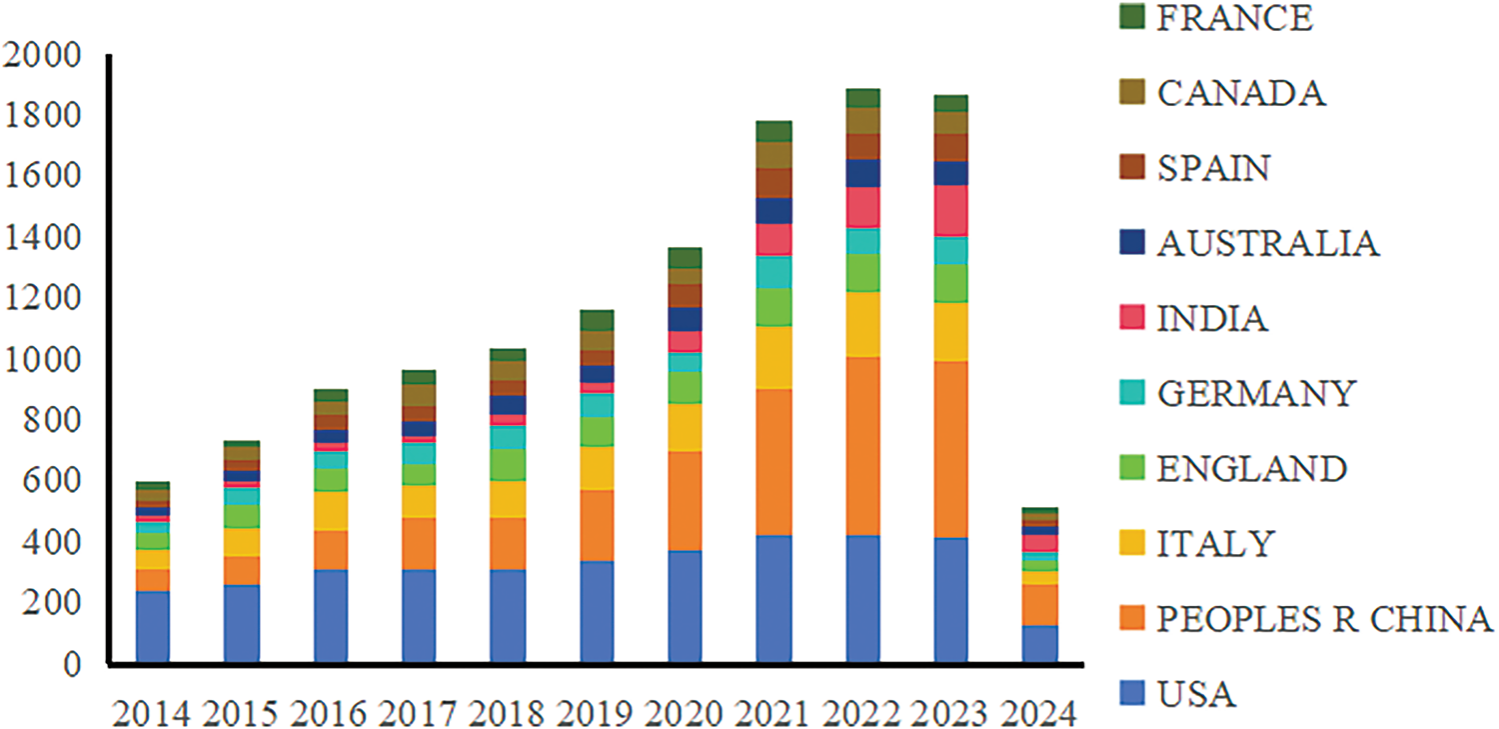
National annual publication trends, 2014–2024.

This study analyzed 17,293 documents from the Web of Science (WOS) English database over the past decade, covering five key areas of cancer research: prevention, screening, diagnosis, treatment, and rehabilitation, as shown in Fig. 2 and Table 1. Specifically, the distribution of articles included 1655 articles on cancer prevention, 2300 on screening, 2874 on diagnosis, 10,298 on treatment, and 166 on rehabilitation. The publication volume peaked between 2021 and 2023. Since the dataset only includes articles from the first two months of 2024, the volume for this year is comparatively low. The top three countries by publication volume were the USA with 3524 articles, China with 2961 articles, and Italy with 1437 articles. Notably, there has been a significant increase in publications from China since 2021, reflecting the growing prominence of its cancer research efforts. However, the importance of translating these research findings into practical applications should also be emphasized.

**Table 1:** National annual publication trends table, 2014–2024 (n > 10)

	USA	CHINA	ITALY	ENGLAND	GERMANY	INDIA	AUSTRALIA	SPAIN	CANADA	FRANCE
2014	237	71	65	57	38	17	27	23	33	34
2015	261	89	91	81	55	20	36	33	43	26
2016	308	129	125	78	60	27	39	49	44	45
2017	311	166	107	72	67	22	54	49	69	45
2018	309	172	115	107	77	36	67	47	61	47
2019	335	239	135	103	74	34	56	55	61	69
2020	374	321	157	108	63	72	76	76	51	67
2021	424	476	205	129	106	101	88	97	86	73
2022	423	587	206	128	85	134	89	86	83	66
2023	414	576	194	128	88	169	81	88	69	61
2024	128	135	37	36	29	56	27	24	19	22

### Distribution of countries/regions

**Figure 3:**
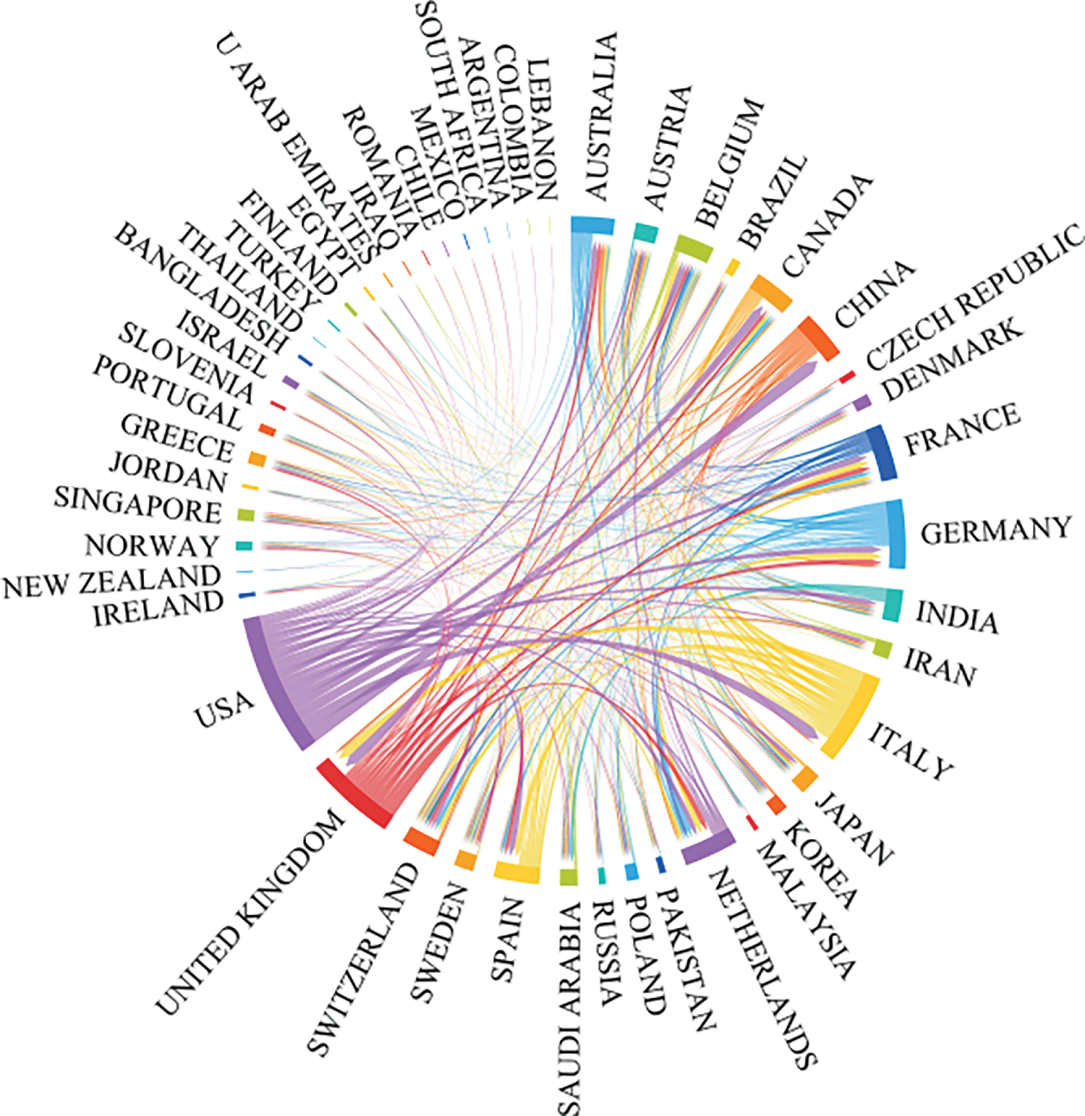
The international collaborations’ visualization map of countries/regions.

**Figure 4:**
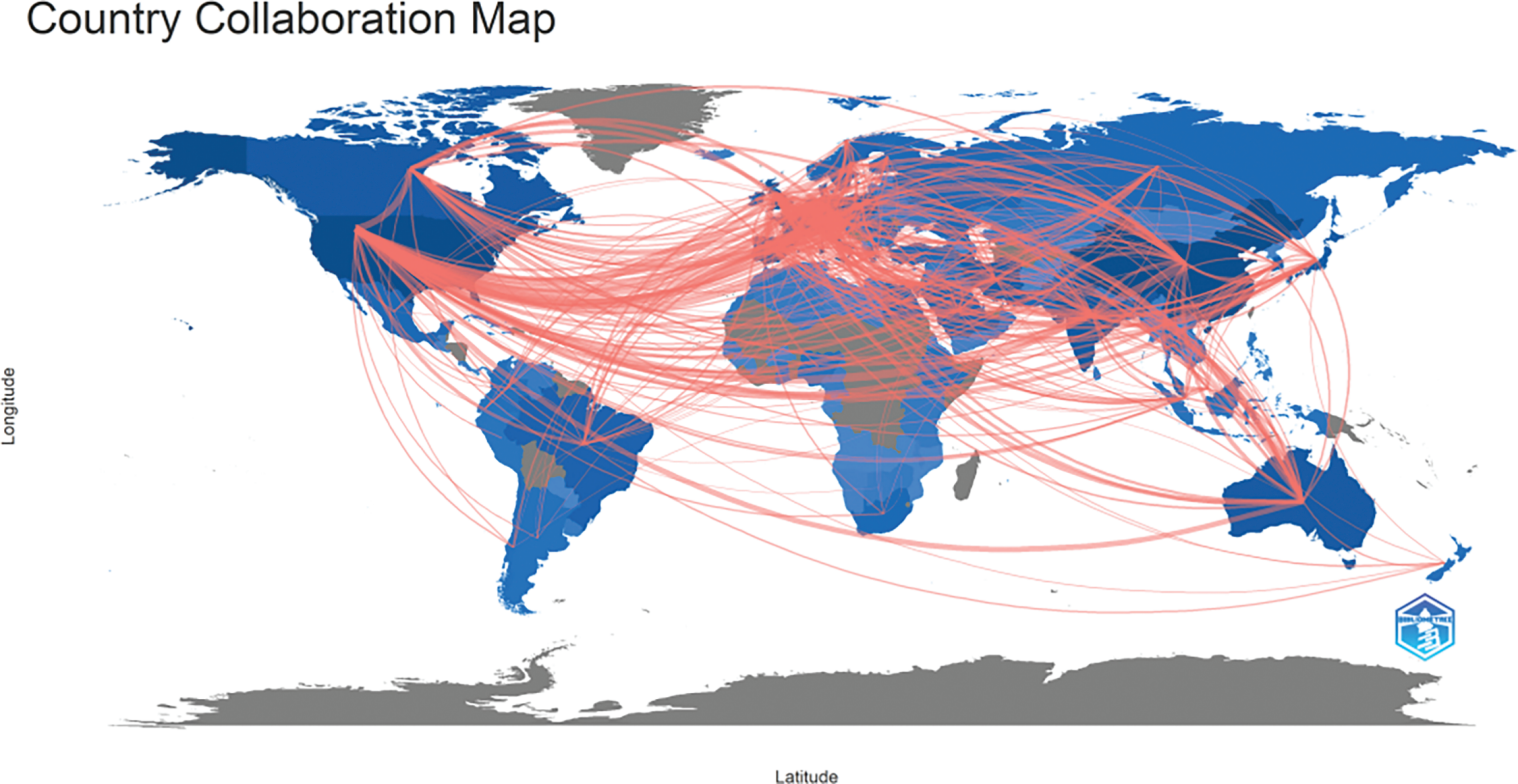
Geographical distribution map based on the total publications of different countries/regions.

International collaboration has played a pivotal role in propelling the advancement of oncology research, achieving remarkable progress via a multidisciplinary approach. Our study reveals that the most fruitful collaboration is between Italy and the USA. Italy actively engages in numerous collaborations with other European countries, a trend facilitated by its geographical proximity to them. Moreover, the USA frequently collaborates with Canada and China, as illustrated in Figs. 3, 4. and Table 2.

**Table 2:** Frequency of inter-country collaboration (n > 10)

From	To	Frequency
ITALY	GERMANY	139
ITALY	SPAIN	132
ITALY	UNITED KINGDOM	189
USA	CANADA	179
USA	CHINA	272
USA	FRANCE	134
USA	GERMANY	140
USA	ITALY	238
USA	SPAIN	123
USA	UNITED KINGDOM	220

### Contributions of institutions

**Figure 5:**
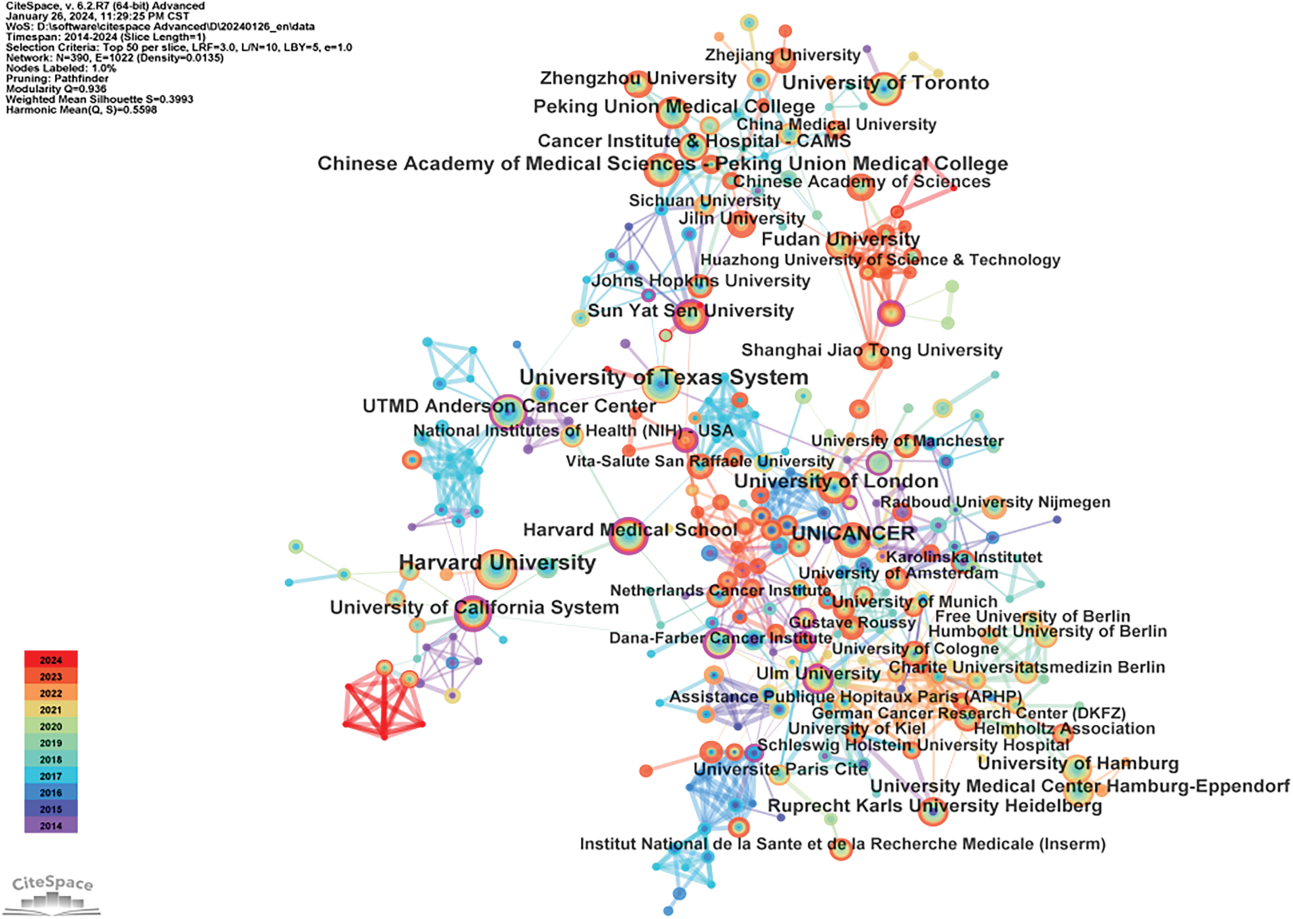
Global institutional collaboration map in the field of oncology.

This study identifies the leading institutions based on publication volume, highlighting the University of Texas, Harvard University, University of London, Peking University Medical School, and the French Federation of Cancer Centers as the top five. Other notable institutions include the University of Toronto, MD Anderson Cancer Center in the USA, and Fudan University, all demonstrating strong capabilities in cancer research. Institutional collaborations are particularly robust within Europe and China, while international collaborations between Chinese and foreign institutions are relatively weak and face certain challenges, as depicted in Fig. 5 and Table 3.

**Table 3:** Frequency table of global institutional collaborations in oncology

Freq	Label	Year
585	University of Texas System	2014
502	Harvard University	2014
394	University of London	2014
392	Chinese Academy of Medical Sciences-Peking Union Medical College	2015
353	UNICANCER	2014
351	University of Toronto	2014
329	UTMD Anderson Cancer Center	2014
313	University of California System	2014
311	Peking Union Medical College	2015
298	Fudan University	2017

### Publication volume and citation analysis of authors

The top contributors in publication volume are Giuseppe Curigliano [4], Anupam Bishayee [5], Amir Avan [6], as detailed in Table 4. Giuseppe Curigliano, MD PhD, ESMO Guidelines Committee Chair, is Full Professor of Medical Oncology at the University of Milano and Chief of the Clinical Division of Early Drug Development at European Institute of Oncology, Milano, Italy. Dr. Curigliano is an expert in advanced drug development for solid tumors, with a specific focus on breast cancer. Dr. Anupam Bishayee primary research interests over the last two decades have included cancer biology, cancer therapeutics, and cancer prevention. His laboratory investigates the mechanism-based chemopreventive and therapeutic effects of various medicinal plants, natural products, dietary agents, and synthetic compounds using diverse pre-clinical cancer models. Amir Avan, PhD, Mashhad University of Medical Sciences, is dedicated to research in cancer biology, immunohistochemistry, and colorectal cancer. Wei Wang, MD, PhD, from Sun Yat-Sen University Cancer Center, is the only Chinese scholar among the top ten in publication volume, specializing in gastric and pancreatic surgery [7].

**Table 4:** Frequency table of author publications in oncology (n > 10)

Frequency	Connections	Centrality	Author	Year
21	9	0.02	Curigliano, Giuseppe	2017
20	15	0	Bishayee, Anupam	2014
18	8	0	Avan, Amir	2017
16	45	0.03	Briganti, Alberto	2015
16	40	0	Fasching, Peter A	2017
15	13	0	Shariat, Shahrokh F	2016
13	44	0	Mueller, Volkmar	2015
13	7	0	Ferns, Gordon A	2018
13	4	0.01	Wang, Wei	2021
12	47	0	Krug, David	2018

**Figure 6:**
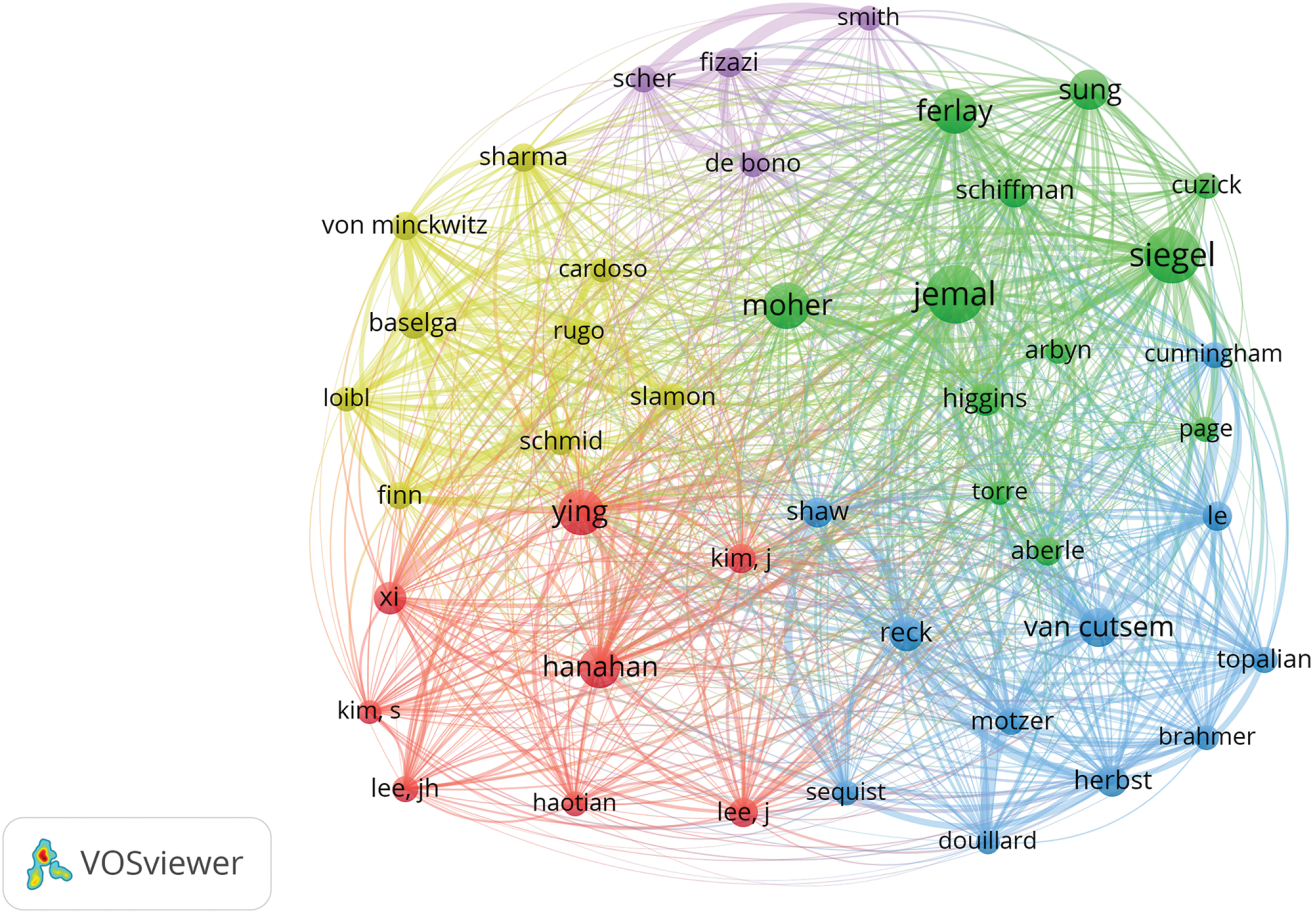
Co-citation network map of authors in oncology.

The frequency table of author publications in oncology (Table 4) highlights the significance of co-citation among authors, where two or more authors are cited together in subsequent articles. This metric helps in identifying high-impact authors within the field. A higher frequency of co-citations between two authors suggests a closer academic relationship, as illustrated in Fig. 6 and Table 5. The top three influential authors are Siegel et al. [8], Siegel et al. [9], and Page et al. [10]. Ahmedin Jemal and Rebecca L. Siegel are noted for their significant contributions like “Global Cancer Statistics,” whereas David Moher specializes in systematic reviews and meta-analyses, including network meta-analysis, and journalology (the science of publication), focusing on reporting guidelines.

**Table 5:** Frequency table of author co-citations in oncology (n > 10)

Frequency	Connections	Centrality	Author
1677	26	0.09	Jemal
1555	12	0.01	Siegel
1059	6	0.02	Moher
954	11	0.04	Ferlay
893	41	0.08	Ying
889	48	0.14	Hanahan
856	36	0.12	Sung
793	12	0.02	Kim
751	25	0.1	Van cutsem
741	42	0.08	Reck

### Journal publication volume and co-citation analysis

This study examines the publication volume across various journals, featuring on those from Q1, Q2, and Q3 JCR categories, with impact factors ranging from 1.6 to 11.8. The three leading journals, all based in Switzerland, are “Cancers” with an impact factor of 5.2, “Frontiers in Oncology” with 4.7, and “International Journal of Molecular Sciences” with 5.6, as indicated in Table 6.

**Table 6:** Frequency table of journal publications in oncology (n > 10)

Journal	Country	Count	IF (2022)	JCR (2022)	Citations	Total link strength
CANCERS	Switzerland	617	5.2	Q2	12,518	1456
FRONTIERS IN ONCOLOGY	Switzerland	371	4.7	Q2	5568	657
INTERNATIONAL JOURNAL OF MOLECULAR SCIENCES	Switzerland	307	5.6	Q1	8816	768
CRITICAL REVIEWS IN ONCOLOGY HEMATOLOGY	USA	160	6.2	Q1	3836	262
MEDICINE	Switzerland	152	1.6	Q3	1609	178
FRONTIERS IN PHARMACOLOGY	Switzerland	130	5.6	Q1	3462	304
CANCER TREATMENT REVIEWS	England	115	11.8	Q1	6031	243
EXPERT REVIEW OF ANTICANCER THERAPY	England	105	3.3	Q3	2025	165
PHARMACEUTICS	Switzerland	99	5.4	Q1	1506	327
MOLECULES	Switzerland	98	4.6	Q2	3451	284

**Figure 7:**
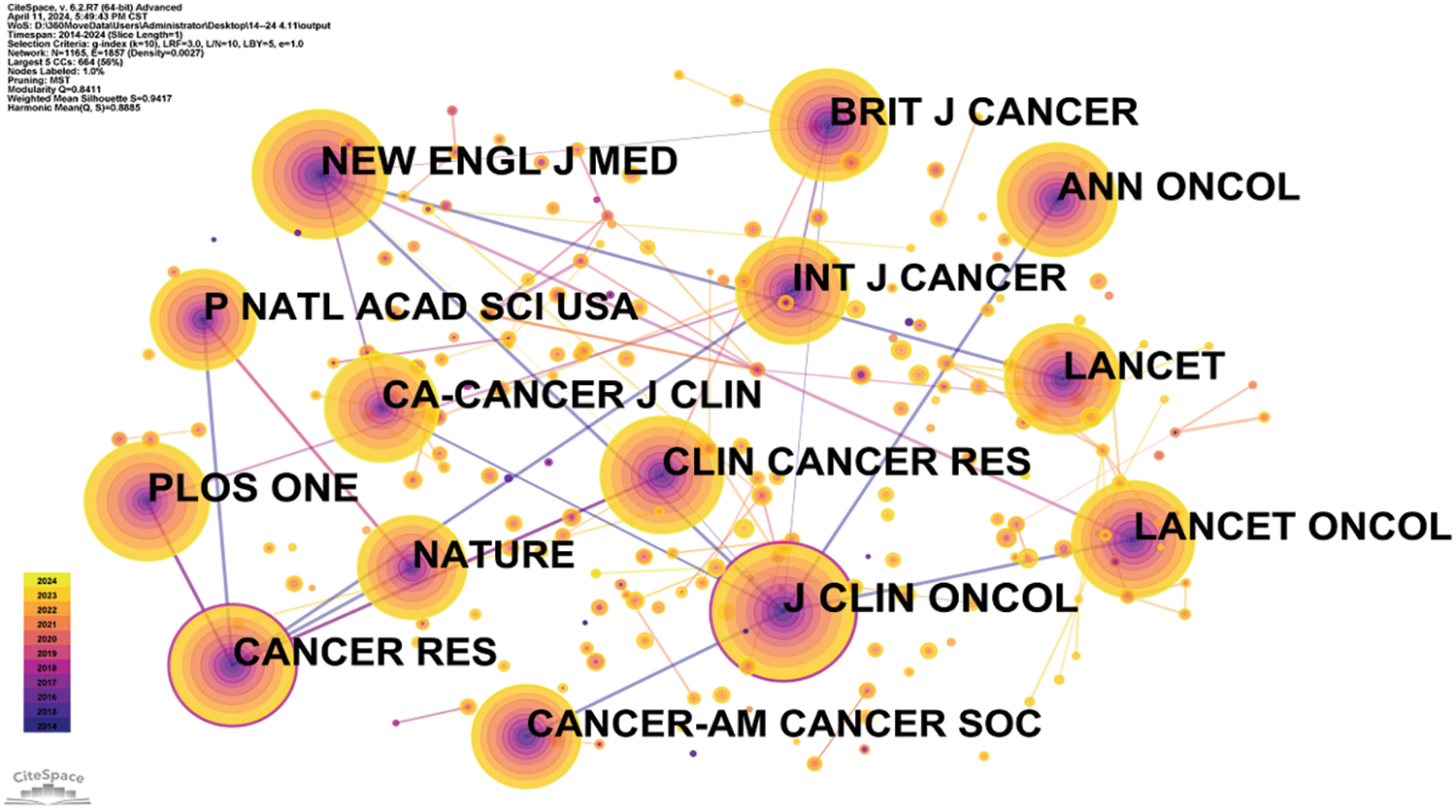
Co-citation network map of journals in oncology.

Journal co-citations serve as a metric for tracking mutual citations between journals, highlighting research trends and key journals in the field. This analysis is illustrated in Fig. 7 and Table 7. The “Journal of Clinical Oncology” leads the rankings in the Q1 category with a 2023 impact factor of 45.3. Other prominent journals include “The New England Journal of Medicine” with an impact factor of 176.07 and the “LANCET ONCOLOGY” with an impact factor of 51.1, both of which are leading publications in oncology.

**Table 7:** Frequency table of journal co-citations in oncology (n > 10)

Co-cites journal	Country	Citations	IF (2022)	JCR (2022)	Total link strength
J CLIN ONCOL	USA	40,696	45.4	Q1	3,842,613
NEW ENGL J MED	USA	23,656	158.5	Q1	2,325,983
CANCER RES	USA	19,515	11.2	Q1	2,921,864
CLIN CANCER RES	USA	18,156	11.5	Q1	2,399,773
LANCET ONCOL	England	14,821	51.1	Q1	1,427,324
ANN ONCOL	Netherlands	14,666	50.5	Q1	1,493,946
PLOS ONE	USA	13,608	3.7	Q2	1,969,178
NATURE	England	12,804	64.8	Q1	1,870,670
P NATL ACAD SCI USA	USA	10,508	11.1	Q1	1,661,395
BRIT J CANCER	England	10,003	8.8	Q1	1,274,972

### Literature co-citation, clustering, and timeline analysis

**Figure 8:**
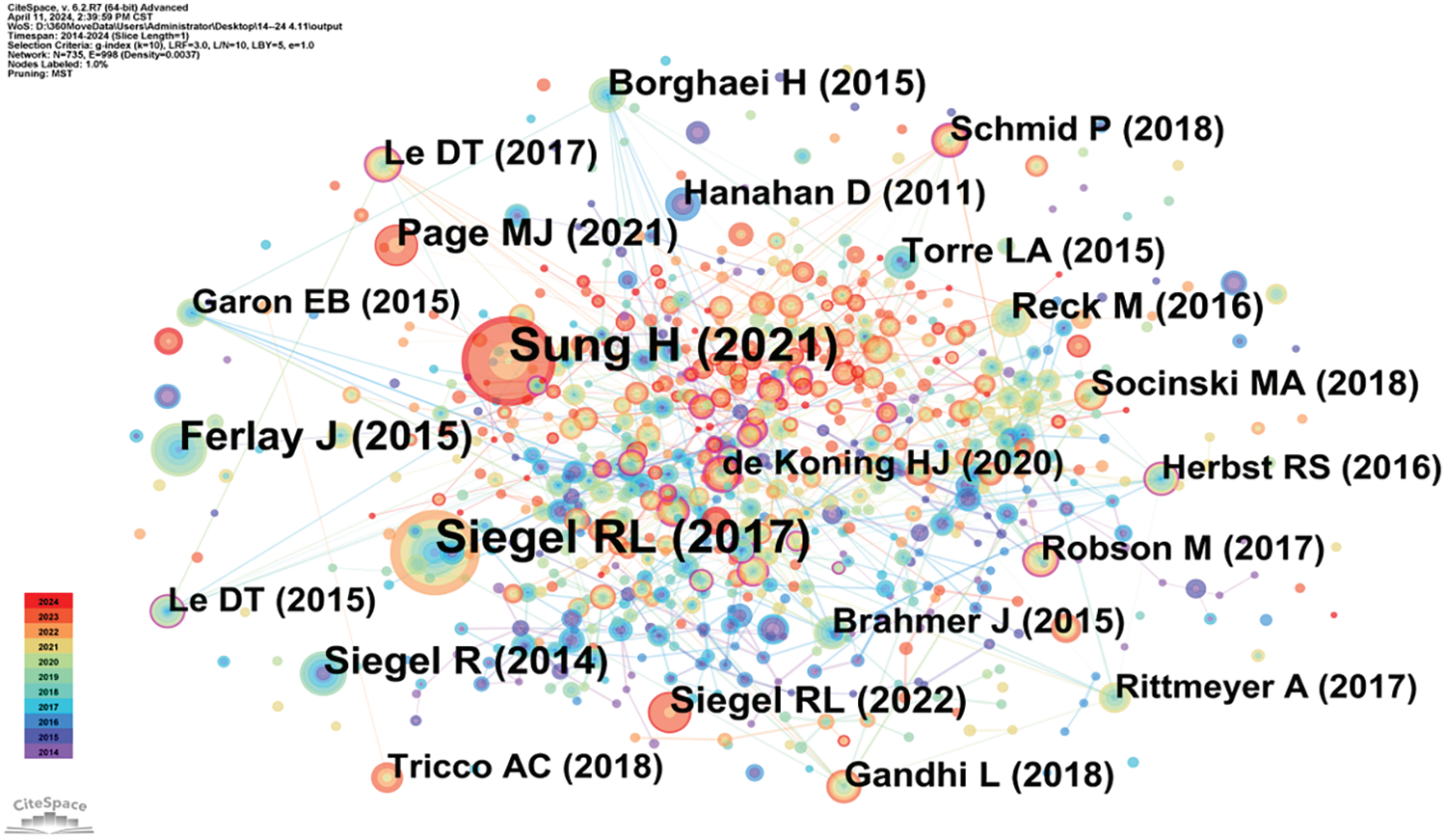
Co-citation network map of literature in oncology.

**Figure 9:**
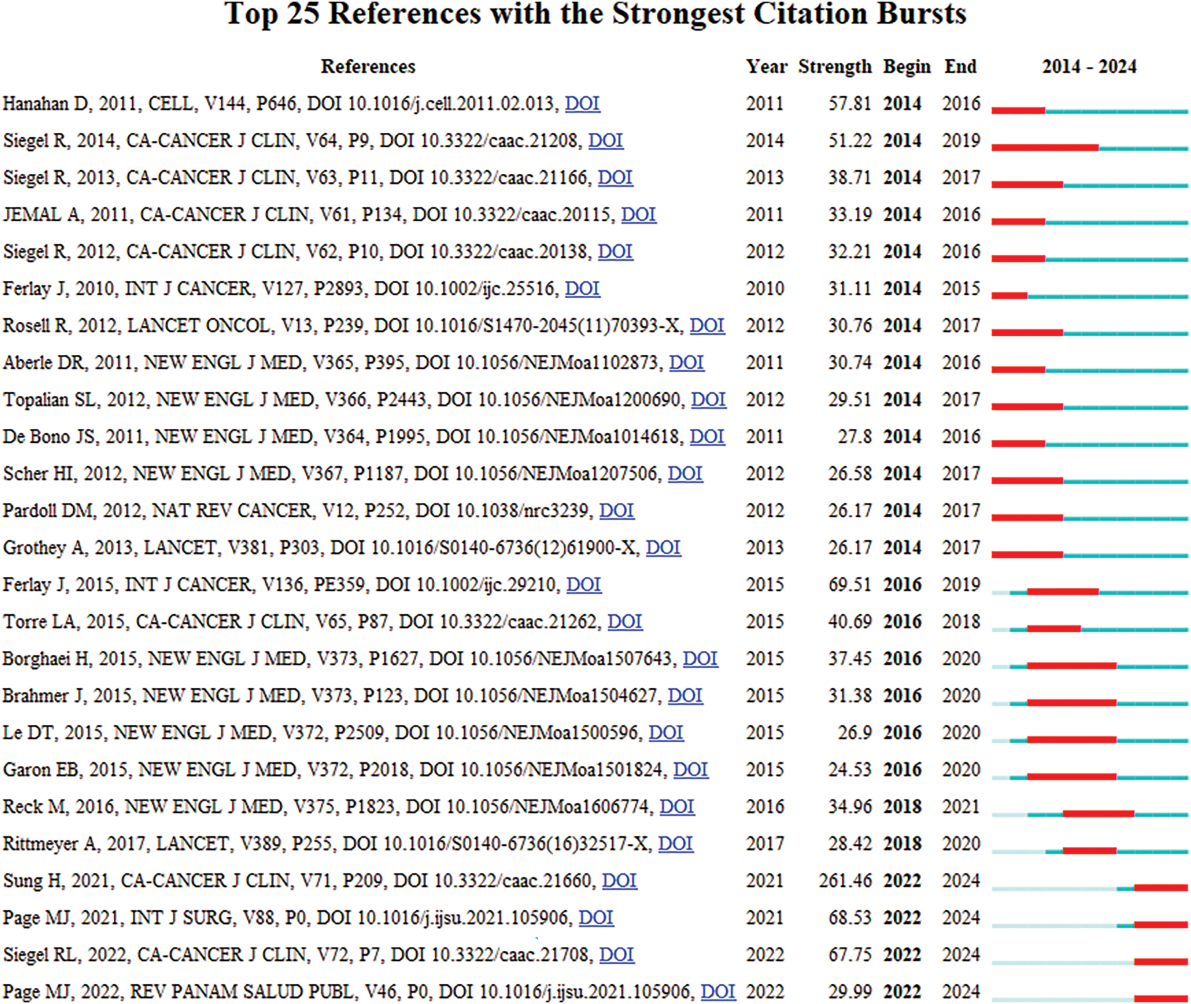
Highlighted citation map of literature in oncology.

Highly cited literature within the field of oncology is of significant interest, encompassing global epidemiological data on cancer and clinical trials over the years, as presented in Table 8 and Fig. 8. Since 2014, there has been an increasing focus in the literature on understanding hallmark features associated with the tumor microenvironment [11], transitioning towards low-dose computed tomography (CT) screening to reduce lung cancer mortality [12], and emphasizing overviews and randomized controlled studies concerning targeted and immunotherapies [13]. These trends reflect the evolving forefront of cancer research, particularly in relation to lung cancer [14], colorectal cancer [15], and metastatic prostate cancer [16], as illustrated in Fig. 9.

**Table 8:** Frequency table of literature co-citations in oncology (n > 10)

Citation frequency	Citation frequency	Year	Publication
789	Sung H	2021	CA-CANCER J CLIN
755	Siegel RL	2017	CA-CANCER J CLIN
300	Ferlay J	2015	INT J CANCER
209	Siegel R	2014	CA-CANCER J CLIN
177	Page MJ	2021	INT J SURG
161	Reck M	2016	NEW ENGL J MED
143	Borghaei H	2015	NEW ENGL J MED
122	Torre LA	2015	CA-CANCER J CLIN
120	Gandhi L	2018	NEW ENGL J MED
120	Brahmer J	2015	NEW ENGL J MED

**Figure 10:**
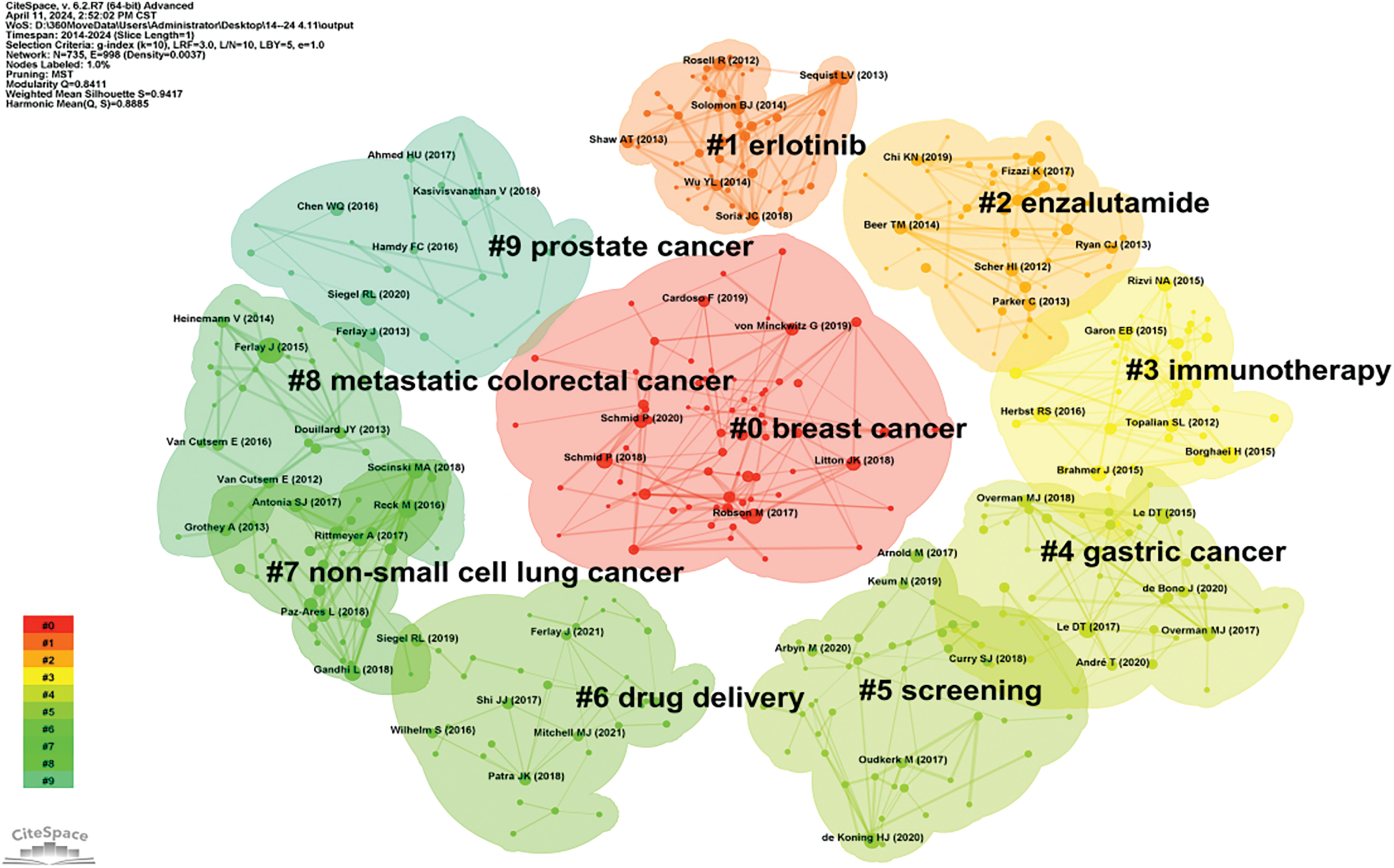
Cluster map of oncology literature.

**Figure 11:**
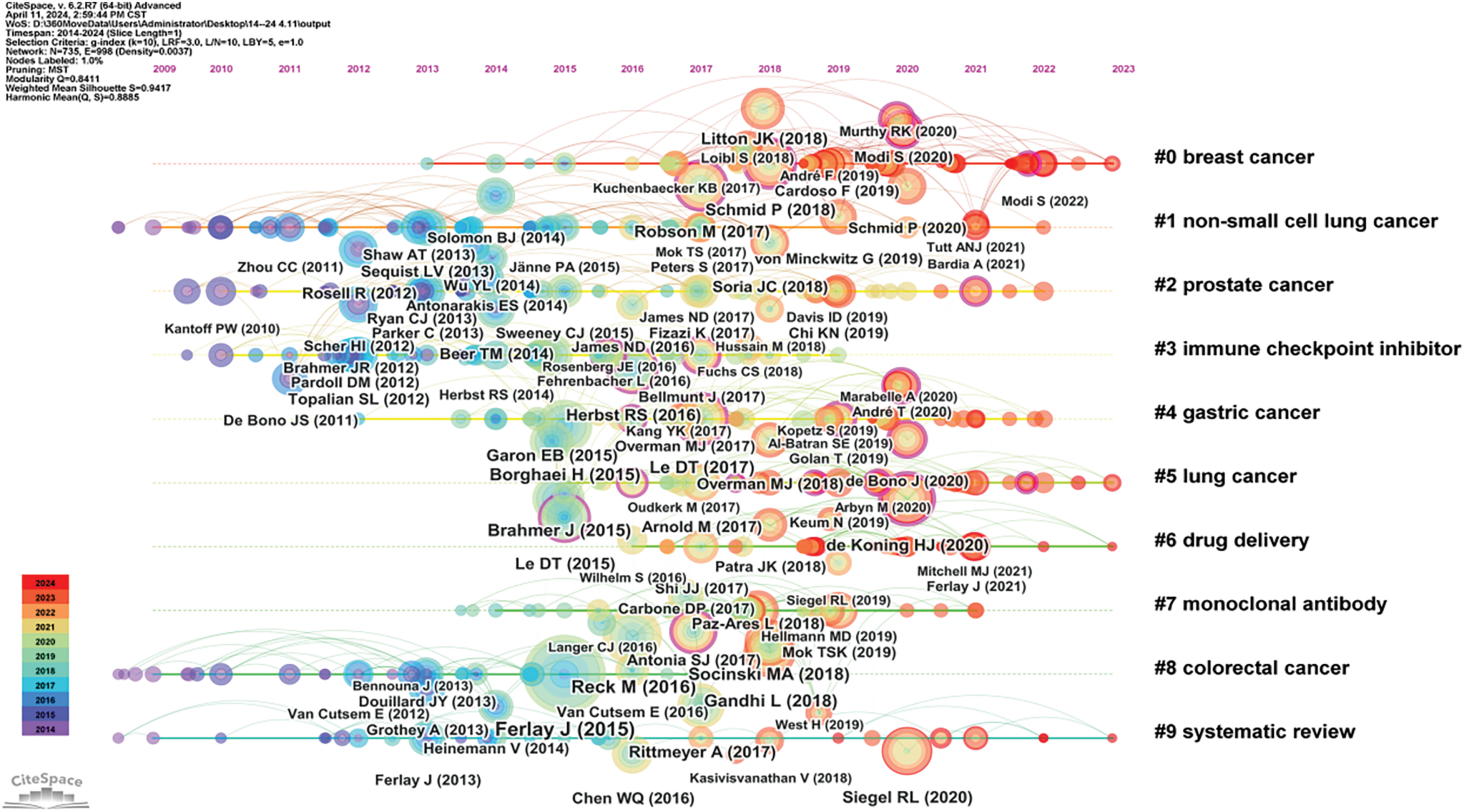
Timeline map of oncology literature.

Clustering and timeline analysis of oncology literature have identified key research areas, including breast cancer, prostate cancer, non-small cell lung cancer, gastric cancer, and metastatic colorectal cancer, as shown in Fig. 10. Over time, trends have shifted towards advancements in immunotherapy, drug delivery systems [17], and the development of monoclonal antibodies [18], as depicted in Fig. 11.

### Keyword analysis

**Figure 12:**
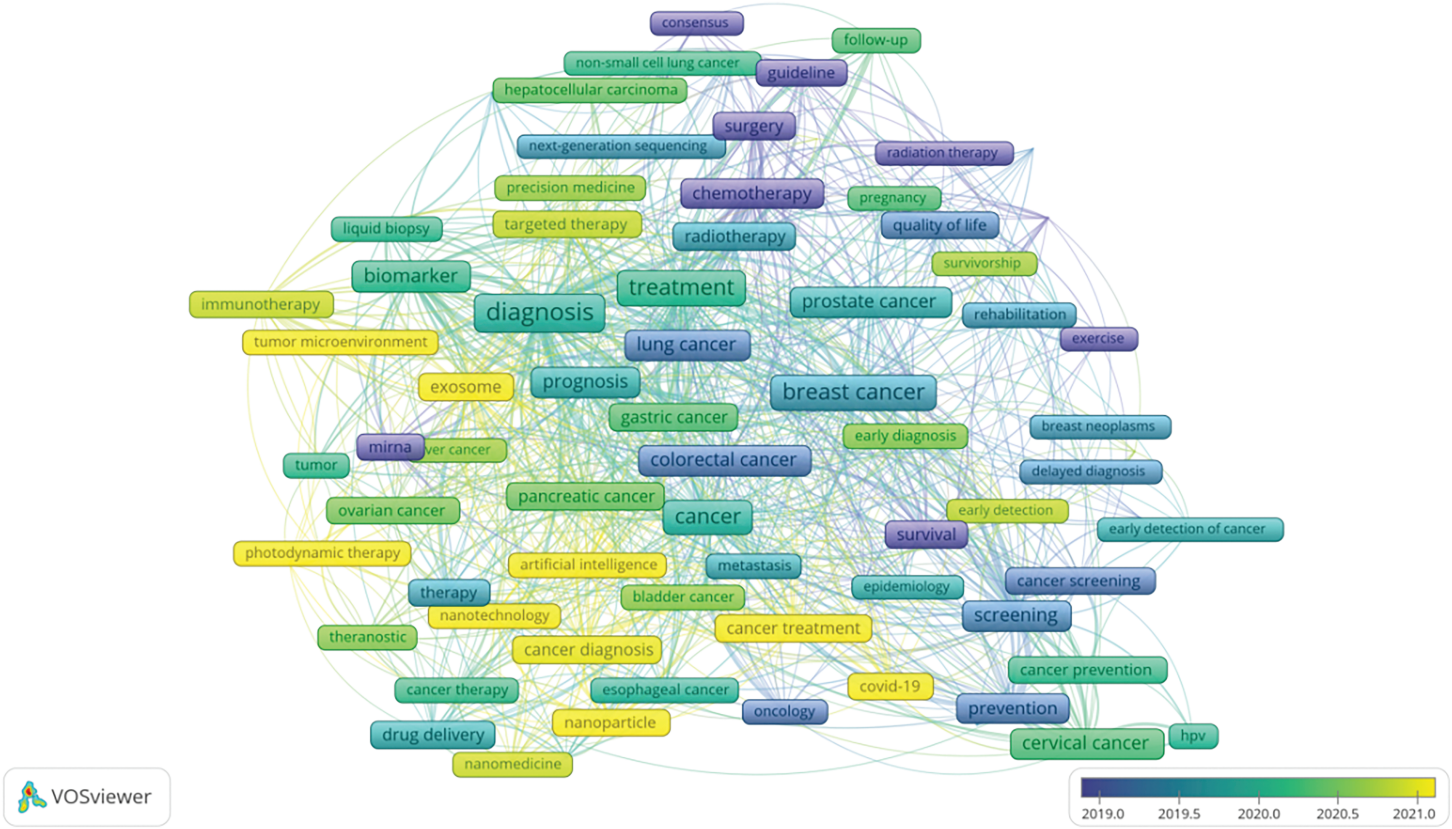
VOS viewer keyword co-occurrence map in oncology.

**Figure 13:**
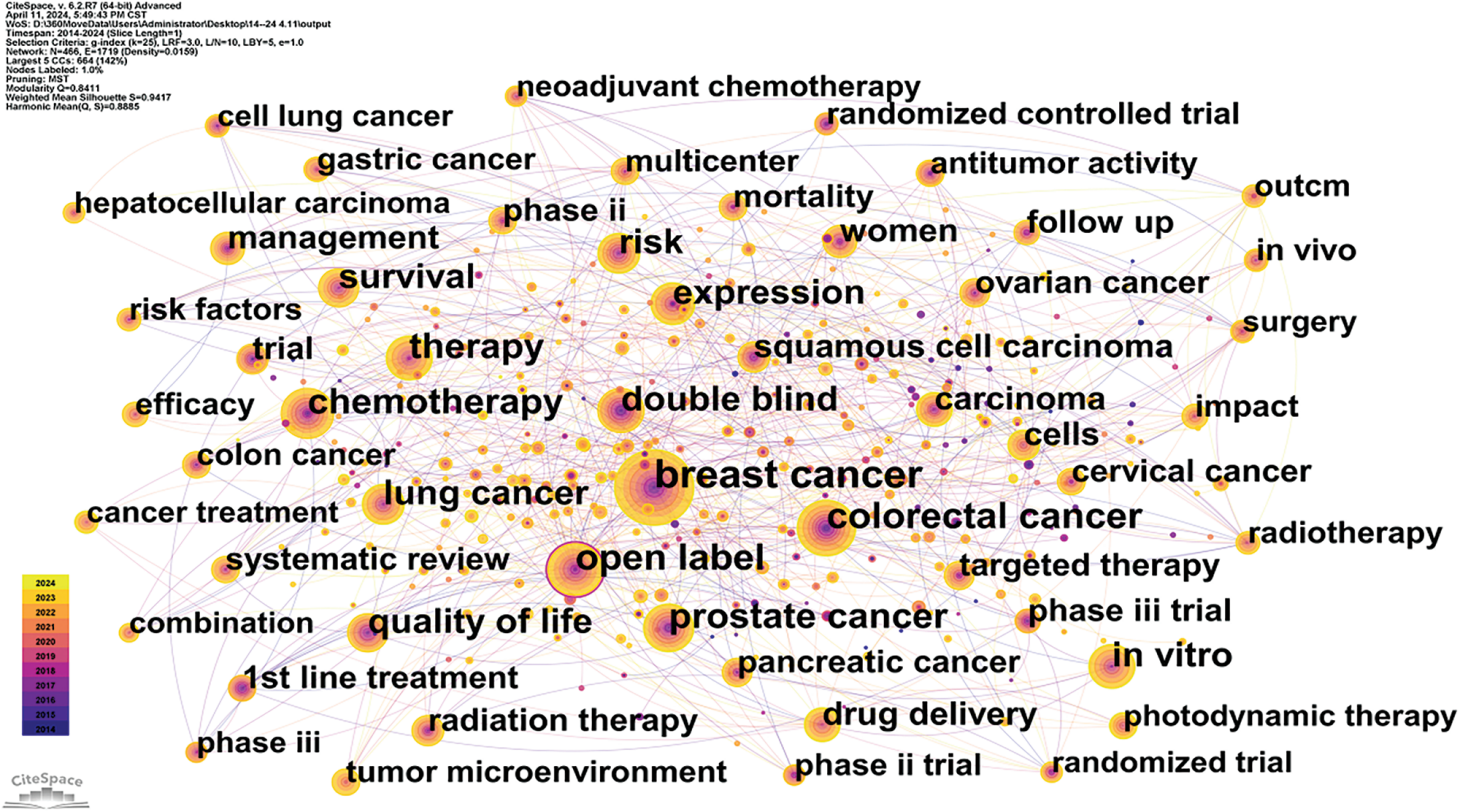
Citespace keyword co-occurrence map in oncology.

Utilizing data from the Web of Science (WOS), this study systematically reviews keywords within the domains of cancer prevention, screening, diagnosis, treatment, and rehabilitation to identify treatment hotspots. Research predominantly focuses on lung and breast cancer [19], with a notable concentration on cancer treatment modalities. Key terms include targeted therapy, immunotherapy, tumor microenvironment, and cancer stem cells [20]. Bibliometric analyses indicate that emerging trends in oncology are likely to include nano-delivery systems, overcoming drug resistance in targeted therapies [21], and the integration of artificial intelligence [22]. A comprehensive overview of keywords in the oncology treatment sector is provided in Figs. 12, 13 and Table 9.

**Table 9:** Frequency table of keywords in oncology (n > 20)

Frequency	Centrality	Keywords	Year
2107	0.04	Breast cancer	2014
1078	0.04	Colorectal cancer	2014
1069	0.12	Targeted therapy	2014
927	0.04	Tumor microenvironment	2017
906	0.02	Gene expression	2014
900	0.02	Immunotherapy	2016
797	0.03	Artificial intelligence	2020
794	0.05	Lung cancer	2014
749	0.03	Double blind	2014
714	0.02	Survival	2014
692	0.06	Neoadjuvant chemotherapy	2014
692	0.01	Cancer stem cells	2018
672	0.02	Quality of life	2014
541	0.04	MiRNA	2014
540	0.1	Drug delivery	2018
481	0.01	Drug resistance	2018
472	0.01	Squamous cell carcinoma	2014
439	0.03	Radiation therapy	2014
418	0.05	Ovarian cancer	2014
417	0.01	Biomarker	2014

## Discussion

**Figure 14:**
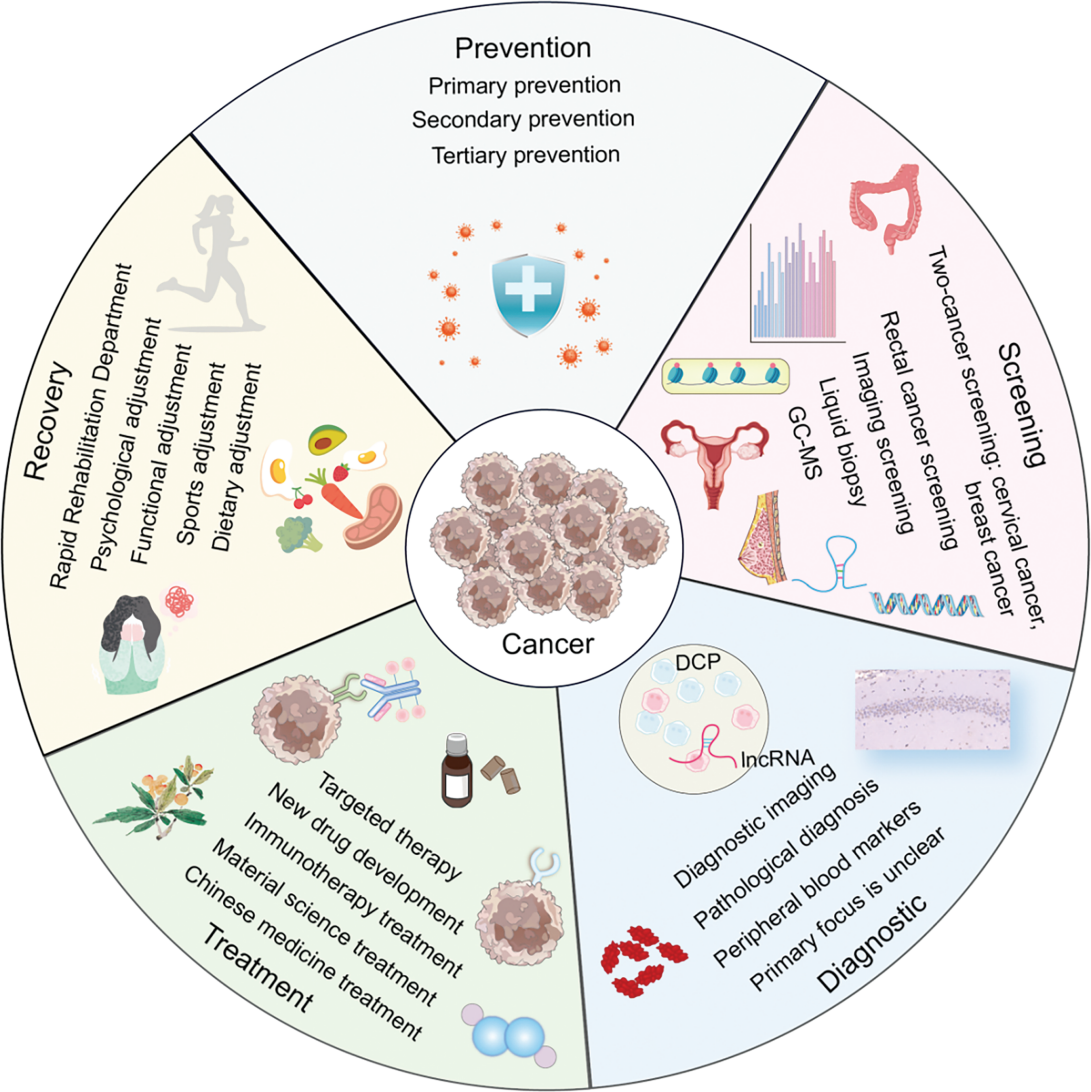
The panorama of cancer prevention, screening, diagnosis, treatment, and rehabilitation (Graphics software: Adore illustrator 2022) (DCP: Des-gamma-carboxy prothrombin, IncRNA: Long non-coding RNA, GC-MS: Gaschromatography-mass spectrometry).

Due to advancements in evidence-based medicine [23] and molecular biology [24], clinical oncology has made rapid progress [25]. We have witnessed the evolution from early treatments of hematologic malignancies [26] to significant breakthroughs in the treatment of solid tumors [27]. This journey spans the initial reliance on radiotherapy [28] and chemotherapy [29] to the advent of molecular targeted therapies [30], immunotherapies [31], and cutting-edge drug research [32]. This shift reflects a move from a traditional, macroscopic understanding of malignant tumors to a more nuanced, microscopic pathological approach of cancer prevention and treatment mechanisms, ultimately, the field has embraced a modern interdisciplinary approach that integrates artificial intelligence [33] and advanced materials [34] to enhance cancer treatment. In light of these developments, we have systematically reviewed a decade’s worth of literature on cancer prevention, screening, diagnosis, treatment, and rehabilitation to provide a comprehensive overview of the current state, emerging hotspots, and development trends in cancer research. The findings are organized for a systematic and exemplary study (Fig. 14).

Keywords serve as a concise summary of a paper’s content [35]. By performing bibliometric co-occurrence analysis of these keywords, it is possible to pinpoint the key research areas within a given field, capturing a comprehensive and cyclical view of research trends and pioneering developments. This approach is particularly invaluable for a holistic understanding of any research area. In the rapidly evolving domain of oncology, bibliometric studies serve as a catalyst, propelling this age-old yet burgeoning field forward. The ongoing scientific and technological revolution plays a pivotal role in advancing cancer research. Delving into the current hotspots, trends, and frontiers of oncology science and technology aids in identifying present challenges, breakthroughs, and future directions in cancer research, offering solutions to the daunting issues of high prevalence and mortality rates associated with cancer.

### Cancer prevention

As China grapples with an aging population, rapid industrialization, and urbanization, and persistent unhealthy lifestyles, the burden of malignant tumors is projected to rise significantly, especially in low and middle-income regions. Over the next two decades, new cancer cases in these regions may increase by as much as 50% [36]. In response to this growing challenge, comprehensive global cancer prevention and control measures are critical. More than 90% of malignant tumors are linked to environmental exposure, lifestyle, and psychosocial factors, with only about 5% attributable solely to genetic susceptibility [37]. Consequently, effective cancer prevention could substantially cancer incidence. The World Health Organization (WHO) classifies cancer prevention into three levels [38]: primary prevention targets risk factors; secondary prevention focuses on early detection, diagnosis, and treatment; and tertiary prevention aims to enhance the quality of life and prognosis for cancer patients. Current scientific evidence suggests that primary prevention could prevent at least 40% of cancer cases [39], while secondary and tertiary prevention strategies will be addressed in subsequent sections of this document. With secondary and tertiary prevention strategies to be discussed further in this document.

Cancer develops through a multi-stage process, where normal cells progressively transform into tumor cells. This transformation typically begins with precancerous lesions and advances to malignant tumors. It is driven by a complex interaction between an individual’s genetic makeup and three main categories of external factors: physical carcinogens, such as ultraviolet and ionizing radiation; chemical carcinogens, including asbestos, tobacco smoke components, alcohol, aflatoxins, and arsenic; and biological carcinogens, such as infections from specific viruses, bacteria, or parasites [40]. Major key risk factors for cancer include tobacco use, alcohol consumption, poor dietary habits, lack of physical activity, and air pollution, among others. Chronic infections are particularly significant in low and middle-income countries and contribute substantially to cancer incidence [41]. In 2018, approximately 13% of cancers globally were linked to carcinogenic infections, such as *Helicobacter pylori* [42], human papillomavirus (HPV) [43], hepatitis B (HBV) and C (HCV) viruses [44], and Epstein-Barr virus (EBV) [45]. HBV and HCV are well-established risk factors for liver cancer, while certain strains of HPV are associated with an increased risk of cervical cancer. Additionally, HIV infection can increases the risk of cervical cancer by a factor of six and elevates the risk of other cancers, including Kaposi’s sarcoma. EBV is notably associated with Burkitt’s lymphoma and nasopharyngeal carcinoma.

Therefore, at the policy level, the “Healthy China Action—Cancer Prevention and Control Action Plan (2023–2030)” [46] underscores the importance of “prioritizing prevention and strengthening the control of cancer risk factors.” This comprehensive plan seeks to establish an integrated management system that covers the entire cancer care process, from prevention and screening of high-risk groups to early diagnosis, standardized treatment, and rehabilitation services. It specifically proposes enhancing cancer prevention and treatment networks at national, provincial, municipal, and county levels, as well as improving quality control systems for tumor diagnosis and treatment. The plan also includes initiatives to promote public health promotion, reduce cancer-related infections, the improvement and promotion of guidelines for the early diagnosis and treatment of key cancers, and further enhancement of the capacity for early screening and early diagnosis and treatment. Although primary prevention has shown some effectiveness in the international field of cancer control, it still faces many challenges, and there is a noticeable gap between its potential effectiveness and practical implementation. Currently, investment in cancer prevention accounts for only 2% to 9% of total cancer research funding. The implementation of primary prevention measures is still relatively slow, and significant effects are only observable after these measures have been in place for a considerable period. There is an urgent need to form a collaborative network that includes the medical services system, public health infrastructure, policymaking bodies, and society at large. As of April 2023, China has established the world’s largest cancer registry system, covering 2806 counties and districts and approximately 1.407 billion people, about 99.8% of the national population. It has selected 1145 national-level cancer registry points, with continuously improving data quality and standardization levels [47].

Building on this foundation, cancer prevention and treatment are advancing towards precision medicine. The etiology of malignant tumors is complex and heterogeneous, with the causes of at least 40% of malignant tumors still unknown. Epidemiological studies investigating the causes of these cancers have been pivotal in uncovering the roles and population distribution of various internal and external risk factors in tumor development. These studies assist in identifying high-risk populations with genetic susceptibility or exposure to carcinogens, and facilitate the creation of individualized malignant tumor risk prediction models. With the advancement of digital technologies, the “Thirteenth Five-Year” national key research and development program by the Ministry of Science and Technology has led to the development of the National Cancer Center’s Department of Prevention and Control’s digital intelligent health management-based cancer primary prevention service and research platform, SmartHMDP-PCP [48]. This platform is designed to address the current challenges in the primary prevention of malignant tumors and enhance the key link in upstream management across the entire cycle and chain of cancer control in China. It also aims to improve cancer prevention education for the population.

From a technological perspective, the integration of AI technology with imaging, pathology, electronic health records, and omics data is promoting the identification of malignant tumor etiology and risk factors, which aids in advancing prevention and enables earlier and more accurate detection and diagnosis of malignant tumors, although it is still in the research and development stage [49]. The implementation of prophylactic surgeries for healthy populations with BRCA gene mutations is feasible but costly, and care must be taken to avoid overtreatment, which may be difficult for the public to accept [50].

The HPV vaccine has proven to be an effective preventive measure against precancerous lesions and related cancers caused by HPV infections [51]. However, it remains expensive, and the vaccines are relatively scarce. Trace elements such as riboflavin have been shown promise in effectively prevent malignant tumors [52]. Despite this, the dissemination of this research remains insufficient, and practical implementation plans are lacking. Chemical drugs like aspirin are known to have a preventative effect on gastrointestinal tumors [53], but their use must be cautious to avoid the adverse effects of overtreatment, including side effects such as bleeding. Traditional Chinese medicine techniques [54], such as health-preserving guidance, have shown effectiveness in preventing tumor occurrence, but they lack robust substantiation from large-scale clinical trials and currently hold a low level of evidence [55]. The journey toward effective cancer prevention remains long and challenging.

### Cancer screening

Identifying key factors that affect cancer screening, which is part of secondary prevention, can significantly enhance early diagnosis rates of tumors. The “Recommendations for Common Malignant Tumor Screening and Prevention” [56] are issued annually by the Shanghai Anti-Cancer Association and the Fudan University Affiliated Cancer Hospital. Current screening protocols include those for cervical and breast cancer. Cervical cancer screening involves gynecological exams, Papanicolaou (Pap) tests, colposcopy, histopathological exams, and HPV testing. Breast cancer screening encompasses clinical breast exams, breast ultrasound, and mammography using X-rays. For lung cancer, screening is conducted using low-dose computed tomography (LDCT) [57]. Colorectal cancer screening involves a preliminary questionnaire combined with a fecal occult blood test (FOBT) and subsequent colonoscopy for follow-up screening, among other methods [58].

Efforts are underway to develop reliable and sensitive detection technologies that can identify cancer cells and their components in body fluids, and to determine their clinical utility [59]. Scholars are conducting extensive studies in this area. In terms of sample testing, early molecular mechanisms—such as changes in abnormal genomes and their transcription products found in the blood or tissues of cancer patients—can serve as early screening markers for specific tumors. For instance, high methylation of tumor suppressor gene promoter regions [60], influenced by carcinogenic factors, can lead to the downregulation or silencing of gene expression, thereby activating the expression of oncogenes and promoting tumor development. Samples commonly used for DNA methylation [61] testing include exfoliated cells, blood samples, and paraffin-embedded tissues. Common detection methods encompass specific polymerase chain reaction (MSP) [62], nucleic acid mass spectrometry, methylation arrays, bisulfite sequencing, and next-generation sequencing technologies [63]. Compared to mutation detection, DNA methylation testing offers several advantages such as high tumor specificity, the capability to detect multiple sites, high signal abundance, and the possibility of tracing back to the tissue of origin. Currently, DNA methylation testing is primarily utilized in clinical applications such as drug guidance for gliomas, adjunctive diagnosis, high-risk stratification in lung cancer, and early screening and recurrence monitoring in colorectal and gastric cancers. Additionally, microRNAs (miRNAs) [64] are abnormally expressed in malignant tumors and play a significant role in the onset and progression of these tumors. In epithelial malignant tumors, miRNAs can be excreted along with exfoliated tumor cells, making the detection of tumor-specific miRNAs in bodily excretions (such as stool, sputum, and urine) a potential new biomarker for the early diagnosis of malignancies in the digestive system, lungs, and bladder [65].

Additionally, circulating tumor DNA (ctDNA) is a type of fragmented DNA in the blood that originates from tumors and serves as a distinctive tumor marker. The detection of ctDNA allows for the identification of tumor traces in a patient’s blood and provides comprehensive data about the tumor within the body, among other benefits [66]. Another method involves using gas chromatography-mass spectrometry (GC-MS) to analyze volatile organic compounds (VOCs) in patients’ exhaled breath enabling the identification and early screening of both benign and malignant lung nodules [67]. However, due to high costs and the lack of large-scale data to verify its accuracy, the widespread adoption of this technology is limited, resulting in a low rate of translation of findings into clinical practice.

In the field of imaging screening, Single-Photon Emission Computed Tomography (SPECT)/CT combines variable-angle dual-probe SPECT with positioning CT [68] to enhance the precision of examinations. Moreover, the integration of artificial intelligence with imaging technologies has spurred the rapid development of radiomics. For example, deep learning radiomics techniques [69], combined with multimodal data (including ultrasound, shear wave elastography, and mammography), are used for the early diagnosis and screening of breast cancer [70].

However, screening programs are effective only for certain types of cancer, not all cancers, and are generally more complex than early diagnosis, requiring more resources. Therefore, screening plans should be specifically targeted based on age and risk factors to minimize the occurrence of too many false-positive results. Additionally, cost-effectiveness assessments should be conducted to establish a reasonable input-output ratio, thus creating a cancer prevention and control management system that is suitable for our country.

### Cancer diagnosis

In addition to early diagnosis, the diagnostic approach in oncology is crucial. Pathological diagnosis remains the gold standard for tumor detection, crucial for initial diagnosis, differential diagnosis, and prognosis. Immunopathology and molecular pathology have broadened the scope of pathological diagnosis from merely morphological observations at the tissue and cellular levels to the protein and molecular levels. For instance, immunohistochemical markers like CK7, CK20, Villin, CDX-2, MUC-2, and PAX8 are employed for the differential diagnosis of ovarian primary mucinous neoplasms (OPMN) and ovarian secondary peritoneal pseudomyxoma [71]. Markers such as INSM1 and CD117, along with traditional neuroendocrine markers, assist in diagnosing lung neuroendocrine tumors, particularly in identifying small cell lung carcinoma [72]. Additionally, the immunohistochemical application of CD34, CD117, BCL-2, and Ki67 proves valuable in diagnosing and assessing the prognosis of breast phyllodes tumors [73].

At the molecular level, peripheral blood markers also play a significant role in tumor diagnosis. Beyond traditional tumor markers, the long non-coding RNA (lncRNA) NEAT1 is abnormally expressed in many malignant tumors and is a significant indicator of poor prognosis. NEAT1 is found to be highly expressed in most malignant tumors, yet current research shows its downregulation in leukemia and renal cancer, suggesting NEAT1’s varying roles across different tumors, which warrants further investigation [74]. Combined diagnostics significantly enhance the accuracy of cancer diagnosis. Des-gamma-carboxy prothrombin (DCP) is a reliable serum tumor marker for diagnosing primary liver cancer (PLC), demonstrating higher sensitivity and specificity than Alpha-fetoprotein (AFP); combining DCP and AFP enhances the diagnostic sensitivity for primary liver cancer and helps avoid clinical misdiagnoses [75]. In non-small cell lung cancer (NSCLC) patients, abnormal expressions of serum miRNA-21, along with Carcinoembryonic antigen (CEA), Cytokeratins 19 (CYFRA21-1), and Neuron specific enolase (NSE) are noted. Elevated serum miR-21 levels have high diagnostic efficiency for early NSCLC and can serve as an important serum biomarker for auxiliary diagnosis [76]. Combined testing with serum CYFRA21-1 levels can further improve diagnostic performance.

In imaging diagnostics, modalities such as Cadmium-zinc-telluride-SPECT (CZT-SPECT) tumor imaging [77], Dynamic contrast enhancement magnetic resonance imaging (DCE-MRI) [78], T1 mapping imaging [79], 18F-fluorodeoxyglucose (18F-FDG) [80], and ^68^Ga fibroblast activation protein PET/CT (^68^Ga-FAPI PET/CT) [81] have significantly enhanced diagnostic accuracy, improved patient comfort during scans, and reduced radiation doses, offering considerable potential in nuclear medicine imaging. Additionally, AI technologies, such as deep learning, convolutional neural networks, radiomics, transfer learning, and image segmentation, can greatly enhance tumor diagnostic accuracy and sensitivity. With adequate training [82], AI can achieve high consistency with the pathological biopsy diagnoses and can also provide hemodynamic parameters, assisting clinicians in differentiating between benign and malignant tumors, thus holding great promise for application in tumor diagnostics.

The diagnosis of cancers of unknown primary origin is also a key research area [83]. Advanced technologies such as gene expression profiling, high-throughput sequencing, epigenetics, and liquid biopsy are increasingly being used to determine the primary site of the cancer, offering high diagnostic accuracy, sensitivity, and specificity. Furthermore, as research into the molecular mechanisms of tumors deepens, the diagnosis and treatment of rare cancers [84], and the identification of rare subtypes combined with pathological and flow cytometric immunotyping, are gradually clarifying diagnostic categories. Although the path is challenging, progress towards a consensus through the translation of research and experience is imminent.

### Cancer treatment

Moreover, Bibliometric analysis of cancer treatment reveals four primary areas of focus: targeted therapy, immunotherapy, traditional Chinese medicine, and the development of novel cancer drugs, mirroring aligning with current trends in the field. In-depth research into gene mutations, immune checkpoint inhibitors, and the tumor microenvironment highlights the present direction in cancer therapy. Emerging frontiers such as nanodelivery systems, artificial intelligence, and resistance mechanisms to targeted therapies signal future breakthroughs in the field of cancer treatment.

Visual analysis reveals that research focus remains on non-small cell lung cancer and breast cancer, the types with the highest incidence and mortality rates. Interest in rare cancers also persists, with research hotspots identified in four key areas: targeted therapy, immunotherapy, traditional Chinese medicine, and the development of new cancer drugs. Since 2018, attention has shifted towards innovations in nanodelivery, artificial intelligence, and overcoming resistance to targeted therapies.

Keyword analysis indicates a move towards a post-chemotherapy era, with chemotherapy research currently accounting for only 14.4% of the field. As a traditional cornerstone of cancer treatment, chemotherapy aims to control or eliminate primary and metastatic tumors, the most significant threats to patients. Yet, the non-selective nature of anticancer drugs towards tumor cells and their toxic side effects have limited chemotherapy’s efficacy. Additionally, the emergence of drug resistance in tumor cells has significantly contributed to chemotherapy’s failure. Advances in molecular biology and genomics have paved the way for the clinical adoption of anti-angiogenic drugs, targeted therapies, and immunotherapies, marking a shift away from conventional chemical treatments. The emphasis on targeted and immune therapies continues to grow, now accounting for 43.8% of research. Cancer targeted therapy specifically addresses molecules or signaling pathways crucial to tumor development and progression. Selecting targeted therapies based on a tumor’s unique molecular characteristics has become the preferred approach for advanced cancer treatments, with new targets continually being identified. The field of antitumor drugs has witnessed numerous breakthroughs, with an annual increase of 5–6 new tumor-targeting drugs. Immunotherapy leverages immunological principles and methods to boost the body’s anti-tumor immune response, effectively killing tumor cells and hindering tumor growth. It offers notable benefits, including efficacy, durability, and minimal toxic side effects. As research advances, an increasing number of previously unidentified genetic mutation targets are expected to be discovered, furthering the development of therapies aimed at well-established targets. The introduction of innovative cancer treatments, such as Antibody-Drug Conjugates (ADCs), holds promise for overcoming existing challenges. The shift toward multidisciplinary treatment strategies, integrating drug delivery systems and artificial intelligence, is emerging as a prominent trend, accounting for approximately 5% of current research. This approach is anticipated to grow, offering solutions to the severe side effects and substantial resistance issues prevalent in past cancer treatments. To gain deeper insights into these evolving research areas, we have performed a detailed keyword clustering analysis on the identified hotspots, with findings as follows:

### Cancer targeted therapy

A keyword analysis of cancer-targeted therapy reveals key focal points in the tumor microenvironment [85], genetic mutations [86], and monoclonal antibodies [87]. Since 2018, there has been a shift toward investigating resistance to targeted therapies [88], tumor stem cells [89], and the application of ADC drugs [90].

**Figure 15:**
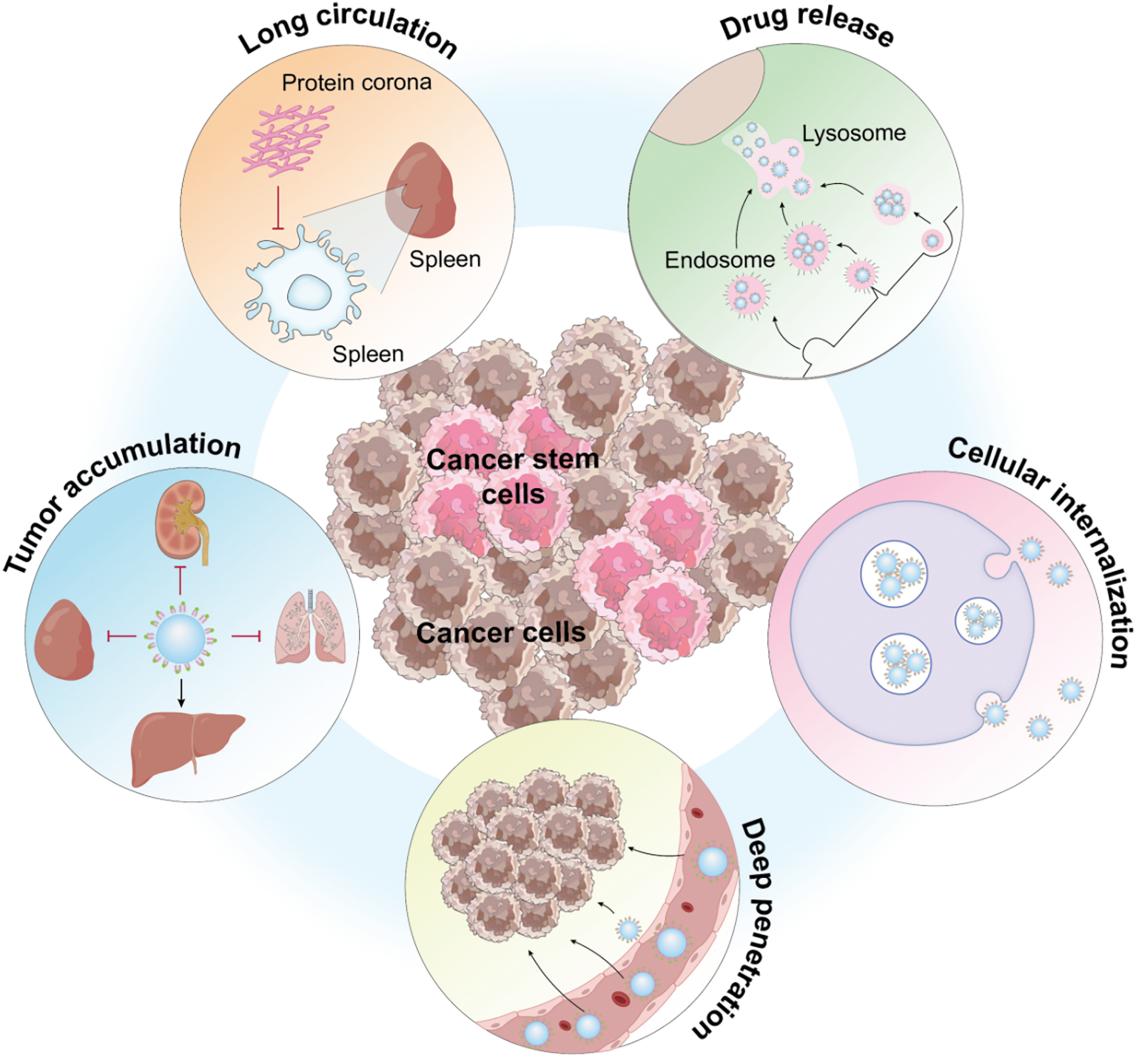
Functional description of Cancer stem cells (Graphics software: Adore illustrator 2022).

The highlighted research areas underscore that the emphasis in targeted therapy research is on exploring therapeutic options for Cancer Stem Cells (CSCs) and the tumor microenvironment. CSCs, a subset of cells within a tumor that exhibit characteristics of both cancer cells and stem cells, are pivotal in tumor recurrence, metastasis, and resistance to treatment. Originating from normal stem cells that undergo the initial carcinogenic mutation, these cells progress to pre-cancerous stem cells and eventually to CSCs [91–93] (Fig. 15). This evolution, fueled by mutations and environmental factors, leads to increased heterogeneity and, coupled with CSC plasticity, drives tumor initiation, progression, and the challenges of recurrence, metastasis, and resistance. Targeted therapy strategies focusing on CSCs have been developed, offering new hope in the fight against cancer. Moreover, the tumor microenvironment, influenced by external factors, including the vascular niche, hypoxia, tumor-associated macrophages, cancer-associated fibroblasts, and the extracellular matrix, are key research focuses in regulating CSCs. Overcoming resistance to targeted drugs remains a significant challenge in future cancer treatments. Resistance mechanisms to small-molecule targeted anticancer drugs are complex, including increased drug efflux, reduced drug uptake, mutations of drug targets, pathway alterations, apoptosis abnormalities, cellular phenotype remodeling, and reactivation of DNA damage repair systems [94,95]. Strategies to counteract these mechanisms involve optimizing drug structures, developing covalent and allosteric inhibitors, employing combination therapies such as ADC drugs [96–98], and targeting proteolytic chimeras. However, as many approaches to tackle tumor drug resistance are in the nascent stages, further clinical evidence is essential for validation.

### Cancer immunotherapy

Visual analysis of the immunotherapy research landscape reveals focal areas such as immune checkpoint inhibitors, mechanisms of immune escape, and the innovation of cancer vaccines. Post-2018, the research has increasingly concentrated on immune-related adverse events and enhancing immune responses.

These key areas indicate that the primary focus of immunotherapy research is on the development of innovative treatments aimed at modulating the tumor microenvironment, develop cancer vaccines, and harness chimeric immunity. Immunotherapy can be categorized into several types, including immune checkpoint therapy, adoptive cell therapy, and cancer vaccine therapy. Within adoptive cell therapy, further distinctions are made among engineered T cell receptor therapy, CAR-T therapy, natural killer (NK) cell therapy, and tumor-infiltrating lymphocyte therapy. While CAR-T cell therapy has demonstrated remarkable success in treating hematologic cancers, its efficacy against solid tumors remains under exploration [99,100]. Cancer vaccine therapy [101,102], which introduces tumor antigens through various means such as cells, peptides, viruses, and nucleic acids, stands out for its low toxicity, high specificity, and potential to generate enduring immune memory. This approach aims to combat tumor heterogeneity and counteract the immunosuppressive tumor microenvironment. Numerous therapeutic vaccines targeting conditions like melanoma, breast cancer, and lung cancer are currently under clinical investigation. Oncolytic viruses [103–105], which selectively infect and destroy tumor cells, represent another innovative avenue. Today’s clinically researched oncolytic viruses, often genetically engineered versions of adenovirus, vaccinia virus, and herpes simplex virus, employ multiple mechanisms to eliminate tumor cells. Oncolytic virus therapy stands out due to its high replication efficiency, effective tumor cell eradication, minimal side effects, and reduced costs, positioning it as a highly promising direction for future cancer immunotherapy. Resistance to targeted therapies remains a major challenge in cancer treatment. Immune-related adverse events (irAEs) can impact various organs, most frequently affecting the skin, gastrointestinal tract, lungs, liver, skeletal muscle, and endocrine system [106–108]. The incidence of these events varies with different drugs, and while less common, adverse reactions can also afflict the nervous system, pancreas, kidneys, hematological system, cardiovascular system, eyes, and other organs. Although the majority of irAEs are mild or moderate (grade 1 or 2), severe adverse reactions (grade 3 or higher) occur in 0.5% to 18.0% of cases, occasionally resulting in patient fatalities. The proactive prediction and prevention of irAEs represent critical areas for future research. Cancer cells can exhibit immune tolerance through mechanisms such as low immunogenicity, impaired antigen presentation, reduced T-cell infiltration, and the upregulation of inhibitory receptors and cytokines. These mechanisms enable them to evade immune cell attacks and prevent the body from mounting a robust tumor-specific immune response, leading to limited effectiveness of tumor immunotherapy in some patients [109]. The immunosuppressive tumor microenvironment contributes to inadequate infiltration and functional exhaustion of effector lymphocytes, significantly diminishing the anti-tumor immune response and rendering the tumor “cold” [110]. This reduces the efficacy of tumor immunotherapy below anticipated levels. Strategies combining various immune checkpoint inhibitors, innate immune activators, and immunoadjuvants, including nanomaterials, show potential in converting “cold” tumors into “hot” tumors, thereby enhancing immune responses and improving therapeutic outcomes. However, these approaches are still under development. Research into precision immunotherapy that extends beyond T cells to include NK cells and dendritic cells is advancing rapidly [111]. NK cells, which can recognize target cells without the need for Major histocompatibility complex (MHC) restriction, exhibit broad anti-tumor efficacy. Their high safety profile in allogeneic environments positions them as highly promising candidates for cancer therapy. For patients with advanced leukemia, NK cell-mediated immunotherapy has emerged as a safe and effective treatment option. Dendritic cells (DCs) play a crucial role in the initial phase of the body’s immune response by recognizing tumor antigens [112]. Upon capturing these antigens, they undergo differentiation and maturation, subsequently presenting antigen signals to immune cells like CD4+ T cells and CD8+ T cells in lymph nodes, thus eliciting antitumor effects. Their potential in treating tumors, particularly solid tumors, is greatly anticipated. Nonetheless, the intricate structural features of the solid tumor microenvironment and the not fully understood mechanisms of DC and T/B cell immune responses pose significant obstacles, hindering major theoretical and technical advancements. M1-type macrophages within the tumor microenvironment display robust antigen-presenting capabilities and contribute to antitumor activity by releasing pro-inflammatory factors and activating type I T cell responses [113–115]. The development of increasingly more immunotherapy strategies targeting tumor-associated macrophages marks a promising area of cancer research.

### Traditional chinese medicine treatment

Research within the realm of Traditional Chinese Medicine (TCM) treatment, as indicated by keyword analysis, currently emphasizes clinical trials, studies on widely used Chinese herbal injections and proprietary Chinese medicines, medication patterns, and the accumulation of expert experience. A trend toward more rigorous methodologies, such as randomized controlled trials (RCTs), the application of hard endpoints in oncology, and investigations into TCM’s role in enhancing quality of life, is becoming evident.

This focus reveals that overall, TCM research remains underdeveloped, particularly in its lack of large-scale RCTs, the gold standard for providing convincing evidence of TCM’s clinical effectiveness. The case of anticancer proprietary Chinese medicines entering the market serves as a case in point: in 2014, these medicines made up 18.15% of the market share. Over the last decade, reports of adverse drug reactions/events related to cancer treatments have seen an average annual growth of approximately 15%, highlighting a significant market presence and usage of cancer medications [116]. However, the widespread claims of efficacy—without sufficient evidence-based support—pose significant challenges for the clinical promotion and application of TCM, underscoring the need for standardized, modern research and development practices. Nevertheless, TCM is gradually aligning more closely with the terminologies and methodologies of modern medicine, such as through investigations into the microscopic mechanisms of action. For instance, heat-clearing and detoxifying agents like *Trametes robiniophila*, *Taxus chinensis*, and Scutellaria barbata [117–120] have been shown to modulate the immune system, counteract immunosuppression, interact with and modify the extracellular matrix and cellular behaviors—including differentiation, proliferation, adhesion, morphogenesis, and phenotypic expression—ameliorate the inflammatory milieu, and interrupt the inflammation-to-cancer progression. Through these multifaceted pathways and targets, such treatments exert a broad regulatory influence by boosting immunity, inhibiting tumor cell growth, promoting tumor cell apoptosis, inhibiting angiogenesis, and encouraging autophagy. Leveraging its inherent strengths and unique attributes, TCM offers distinct advantages in managing symptom clusters, notably enhancing cancer-related fatigue, and mitigating the adverse effects of conventional cancer treatments such as chemotherapy, radiotherapy, targeted drug therapy, and immunotherapy [121,122]. It provides relief from symptoms like rashes, nausea, and vomiting, thereby improving the quality of life for cancer patients. Additionally, acupuncture, recognized for its capacity to supplement deficiencies, eliminate excesses, harmonize Yin and Yang, and clear meridians, utilizes acupoint stimulation to regulate body functions, dispel pathogenic factors, and bolster healthy energy. Modern medical research, including clinical observations, cytology, and molecular biology studies, has demonstrated that acupuncture can modulate the immune microenvironment, boost the body’s immunity, suppress tumor growth, and ameliorate clinical symptoms in cancer patients [123–125]. Given its minimal invasiveness and low cost, acupuncture holds significant clinical relevance for cancer treatment and warrants broader clinical adoption.

### New cancer drug development

In the realm of cancer drug development, the focus is increasingly shifting towards targeting tumor stem cells, gene regulation, and small RNAs, employing cutting-edge techniques such as organoid technologies, artificial intelligence (AI), and 3D printing [126]. The new therapeutic agents being developed predominantly include monoclonal antibodies, bispecific antibodies, and Antibody-Drug Conjugates (ADCs), with post-2018 trends highlighting an emphasis on organoid models, nanomaterials, and AI applications.

**Figure 16:**
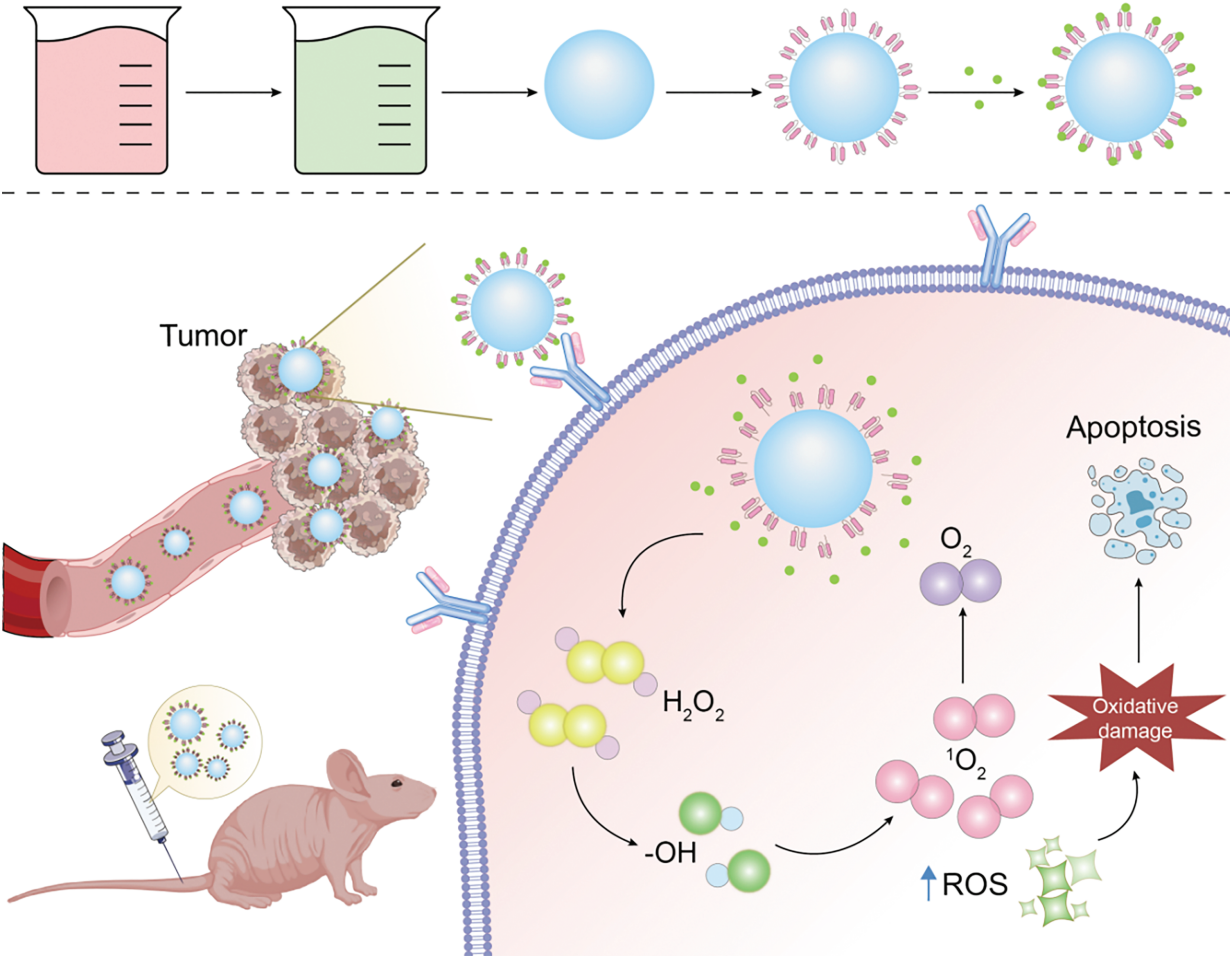
Nano-delivery mechanism diagram (Graphics software: Adore illustrator 2022).

This analysis of new drug development hotspots underscores that progress in this field is increasingly driven by novel insights into tumor biology, which in turn inform the identification of drug targets. Targeting specific tumor stem cell markers, signaling pathways, microenvironments, and metabolic pathways has shown promise in effectively inducing tumor cell apoptosis, pointing to a future where cancer treatment is both more precise and effective. Several novel drugs targeting tumor stem cells, including Epigallocatechin Gallate [127], MK-2206 [128], and Navitoclax [129], have demonstrated promising results in clinical trials. However, the body of clinical data supporting their efficacy is still growing. Looking ahead, cancer treatments designed to specifically target the heterogeneity of a patient’s tumor stem cells—and to eradicate precancerous stem cells and their progeny—hold great promise for curbing tumor growth, metastasis, and recurrence. Furthermore, the innovation in new drug types is intricately linked to advancements in biosynthesis. Synthetic biology [130–133] through the discovery, comprehension, and reengineering of molecular components, facilitates the creation of novel biological functions. It enables the synthesis of bacteria, gene circuits, proteins, carbohydrates, nucleic acids, viruses, and multicellular systems. For example, using genetically engineered bacteria designed through synthetic biology to function in hypoxic conditions allows these organisms to produce cytotoxic proteins specifically in the oxygen-poor tissue environments of tumors, thereby triggering cancer cell death and effectively targeting cancer cells. While synthetic biology holds vast potential, its application is currently primarily confined to laboratory research. In the foreseeable future, its translation into clinical applications, including bacterial therapies for cancer, cell therapy, and cancer vaccines, is highly anticipated. Lastly, the landscape of new drug types is also evolving. Nanotechnology [133–135] has emerged as a crucial tool for addressing the challenges of targeted drug delivery, and the advancement of nanotechnology-based delivery systems marks a groundbreaking development in the field of oncology (Fig. 16). The use of nanoparticles to deliver and control therapeutic agents offers several advantages, such as targeted delivery, controlled release, biocompatibility, low toxicity, and acceptable degradation rates. This approach significantly improves the bioavailability of chemotherapy drugs, reducing their adverse effects and facilitating efficient, durable, and minimally side-effective treatment outcomes. Moreover, by increasing the lipophilicity of nanomaterial surfaces and reducing their surface charge, their ability to cross the blood-brain barrier can be improved, offering a promising strategy for targeting brain metastases. However, the application of nanomaterials in tumor treatment remains in its developmental stages. Challenges persist in controlling the metabolism and distribution of nanomaterials within the human body, and conclusive evidence regarding their safe metabolism without toxic accumulation is still lacking. Additionally, the biocompatibility of nanomaterials warrants further investigation. The compatibility of nanomaterials with the human body and their potential interactions with other substances, possibly leading to adverse effects, is yet to be fully understood. Another unresolved issue is the determination of safe dosages for nanomaterial-based drugs, as no standardized guidelines currently exist. Despite these challenges, the transition from development to application is an ongoing process. With the rapid advancements in AI in recent years, an increasing number of research institutions and companies are leveraging AI for drug development. Looking forward, AI holds the potential to preemptively predict resistance mechanisms of small molecule targeted anticancer drugs and propose strategies to counteract such resistance. The future of AI in pharmaceutical development, particularly in addressing tumor resistance, is filled with optimism.

### Cancer rehabilitation

Moreover, cancer patients frequently encounter a range of challenges, including psychological disorders, functional impairments, nutritional disturbances, physical disabilities, and barriers to social reintegration, underscoring the significant potential for cancer rehabilitation interventions. This field can be segmented into various stages such as the diagnostic period, perioperative period, chemotherapy/radiotherapy period, and terminal stage. Rehabilitation strategies include psychological support, pain management, physical functional rehabilitation, nutritional support, and more.

For example, studies have shown that pulmonary rehabilitation significantly enhances the quality of life for lung cancer patients after surgery and chemotherapy. This rehabilitation includes pain management, psychological care, and exercises like chest expansion with deep breathing, which improves ventilatory function, lower limb rehabilitation, and respiratory training. Results indicate that after 12 weeks of such exercise, patients show improvements in bodily functions, reduced postoperative complications, fewer adverse reactions, better overall quality of life, and reduced anxiety, particularly in terms of fatigue, pain, breathing difficulties, and insomnia, thereby demonstrating that rehabilitation training can significantly improve patient quality of life [136].

Additionally, intestinal rehabilitation for lung cancer patients suffering from chemotherapy-induced constipation has shown marked improvements in both bowel function and emotional well-being, further improving quality of life [137]. Mindfulness cancer rehabilitation training combined with auricular acupressure care for colorectal cancer chemotherapy patients has effectively reduced gastric discomfort and cancer-related fatigue, improving self-efficacy and quality of life [138]. Dietary interventions using medicinal foods have also been found to improve the nutritional status of patients with nasopharyngeal cancer undergoing radiotherapy [139].

The publication of guidelines and expert consensus documents, such as the ‘Consensus on Nutritional Management During the Rehabilitation of Malignant Tumor Patients’ [140] and the ‘Consensus on Exercise Rehabilitation Focused on Functional Impairment in Cancer Patients in China’ [141], provides a robust foundation for clinical decision-making, using well-established and standardized outcomes for oncological rehabilitation in current practice.

Fast Track Surgery (FTS) [142,143] refers to the implementation of optimized, evidence-based approaches before, during, and after surgery to minimize physiological and psychological trauma in the perioperative period, thereby facilitating rapid recovery. Introducing the concept of fast track surgery in perioperative care for gastric cancer surgery patients has been shown to effectively accelerate gastrointestinal functional recovery post-surgery, prevent complications, alleviate surgical pain, significantly reduce hospital stay lengths, and lower treatment costs.

In addition to its antitumor effects, TCM has demonstrated significant benefits in improving the quality of life for cancer patients during chemotherapy intervals and rehabilitation. It alleviates myelosuppression during chemotherapy intervals, significantly inhibits the decline of peripheral blood cells, and protects liver and kidney functions. Wang and colleagues [144] conducted a multi-center, large-sample cohort study where the treatment group received a comprehensive treatment regime that included differential diagnosis herbal soup, exercise, psychological support, dietary adjustments, functional nourishment, and ointment therapies. The study found significant improvements in anxiety and depression. Furthermore, the efficacy of the “Yifei Qinghua Ointment” was analyzed in the treatment group, where it was administered orally alongside conventional Western medicine [145]. The treatment group exhibited significant improvements in Karnofsky performance scores, weight changes, and immune function compared to the control group, with no severe adverse reactions. This approach also demonstrated good efficacy in combating cancer-related fatigue, cancer-associated diarrhea, and cancer-related constipation, thereby improving quality of life scores.

## Conclusion

The past decade has seen significant advancements in evidence-based medicine and molecular biology, which have driven notable progress in the field of malignant tumors. We have seen progress from the early treatments of hematological malignancies to breakthroughs in solid tumors; from the initial use of radiotherapy and chemotherapy to the evolution of molecular targeting and immunotherapy, and further into the research of new drugs. This evolution reflects a shift from a macroscopic understanding of malignant tumors to in-depth investigations of their mechanisms at the microscopic level, and has increasingly incorporated multidisciplinary strategies, such as artificial intelligence and materials science, to modernize cancer treatment.

The current research status in cancer prevention, screening, diagnosis, treatment, and rehabilitation remains a focal point of discussion, with emerging areas such as nanocarrier drug delivery, targeted drug resistance, and immune vaccines still in the research stages indicating future developmental trends. In the near future, the rate of translation of research findings into practice will continue to improve. The challenges currently faced in managing malignant tumors will be addressed, and milestone advancements will be achieved, setting the stage for the next generation of cancer research and ushering in a new era of cancer treatment.

Limitations

Due to the limitations of the search criteria and software, only literature from the Web of Science Core Collection was included, which may introduce some bias in the results. Additionally, non-English publications were not incorporated. In future research, we will conduct a detailed analysis of the five major areas of malignant tumor prevention, screening, diagnosis, treatment, and rehabilitation to gain a comprehensive understanding of the field. Furthermore, since the field of cancer rehabilitation is an underdeveloped area, the number of studies included is smaller compared to the treatment field. We will continue to supplement and track the latest developments in the full-cycle cancer field in subsequent studies.

## Data Availability

Not applicable.
